# Null results of oxytocin and vasopressin administration on mentalizing in a large fMRI sample: evidence from a randomized controlled trial

**DOI:** 10.1017/S0033291721004104

**Published:** 2023-04

**Authors:** Mark A. Straccia, Adam R. Teed, Perri L. Katzman, Kevin M. Tan, Michael H. Parrish, Michael R. Irwin, Naomi I. Eisenberger, Matthew D. Lieberman, Benjamin A. Tabak

**Affiliations:** 1 Department of Psychology, University of California, Los Angeles, CA, USA; 2 Department of Psychology, Southern Methodist University, Dallas, TX, USA; 3 Department of Psychology, New York University, New York, NY, USA; 4 Department of Psychiatry and Biobehavioral Sciences, David Geffen School of Medicine, University of California, Los Angeles, CA, USA; 5 Cousins Center for Psychoneuroimmunology, Jane and Terry Semel Institute for Neuroscience, David Geffen School of Medicine, University of California, Los Angeles, CA, USA

**Keywords:** fMRI, functional connectivity, mentalizing, oxytocin, theory of mind, vasopressin

## Abstract

**Background:**

Although potential links between oxytocin (OT), vasopressin (AVP), and social cognition are well-grounded theoretically, most studies have included all male samples, and few have demonstrated consistent effects of either neuropeptide on mentalizing (i.e. understanding the mental states of others). To understand the potential of either neuropeptide as a pharmacological treatment for individuals with impairments in social cognition, it is important to demonstrate the beneficial effects of OT and AVP on mentalizing in healthy individuals.

**Methods:**

In the present randomized, double-blind, placebo-controlled study (*n* = 186) of healthy individuals, we examined the effects of OT and AVP administration on behavioral responses and neural activity in response to a mentalizing task.

**Results:**

Relative to placebo, neither drug showed an effect on task reaction time or accuracy, nor on whole-brain neural activation or functional connectivity observed within brain networks associated with mentalizing. Exploratory analyses included several variables previously shown to moderate OT's effects on social processes (e.g., self-reported empathy, alexithymia) but resulted in no significant interaction effects.

**Conclusions:**

Results add to a growing literature demonstrating that intranasal administration of OT and AVP may have a more limited effect on social cognition, at both the behavioral and neural level, than initially assumed. Randomized controlled trial registrations: ClinicalTrials.gov; NCT02393443; NCT02393456; NCT02394054.

## Background

A number of oxytocin (OT) and vasopressin (AVP) administration studies point to a possible role for these neuropeptides in improving mentalizing (Brunnlieb, Münte, Tempelmann, & Heldmann, 2013; Domes, Heinrichs, Michel, Berger, & Herpertz, 2007; Tomova, Heinrichs, & Lamm, 2019), the ability to infer and represent others' mental states (Frith & Frith, 1999). Based on results such as these, one of the primary goals of research in this area has been to use OT or AVP as a treatment for individuals with social cognitive impairments (Quintana et al., 2021). However, it is currently unclear whether OT or AVP specifically influences mentalizing. There has been a lack of replication for the behavioral effects of OT and AVP on a range of social processes (Tabak et al., 2019), including mentalizing (Radke & de Bruijn, 2015). In addition, most studies have included all-male samples despite numerous sex-specific effects (Quintana et al., 2021), and surprisingly few have investigated the effects of OT or AVP specifically on mentalizing.

The majority of these studies include the Reading the Mind in the Eyes Test (RMET; Baron-Cohen, Wheelwright, Hill, Raste, & Plumb, 2001), in which participants identify emotional states from only the eye region of face stimuli. A recent meta-analysis of OT administration studies, many of which included the RMET, found that OT did not affect theory of mind in healthy or clinical samples (Leppanen, Ng, Tchanturia, & Treasure, 2017). However, several studies using the RMET have demonstrated moderation of OT effects by person-specific factors (Feeser et al., 2015; Luminet, Grynberg, Ruzette, & Mikolajczak, 2011; Riem, Bakermans-Kranenburg, Voorthuis, & van IJzendoorn, 2014; Schwaiger, Heinrichs, & Kumsta, 2019; Sun, Vuillier, Deakin, & Kogan, 2020). Importantly, although the RMET is typically conceptualized as a mentalizing task, there is some ambiguity in this classification as others have viewed it as an emotion recognition task (Adolphs, Baron-Cohen, & Tranel, 2002; Uzefovsky, Shalev, Israel, Knafo, & Ebstein, 2012), and deficits in RMET performance have been disassociated from theory of mind performance (Oakley, Brewer, Bird, & Catmur, 2016). Emotion recognition has been viewed as a process distinct from, overlapping with, or even subsumed by mentalizing, which shares a nearly identical definition with theory of mind, and is typically associated with cognitive rather than emotional mental state inferences (Mitchell & Phillips, 2015).

A few studies have examined the effects of OT on other mentalizing tasks. In healthy control groups, one study (*n* = 25) found no effects of OT in the False-Belief Task, which involves mentalizing in the context of a hypothetical scenario (De Coster, Lin, Mathalon, & Woolley, 2019), and another (*n* = 31) found no effect of OT using The Awareness of Social Inference Test (Woolley et al., 2014), which elicits social inferences from video clips of individuals in contexts of varying complexity (McDonald et al., 2006). Two studies of healthy individuals (*ns* = 48–59) used the Multifaceted Empathy Test (Dziobek et al., 2008) to distinguish between mentalizing, or cognitive empathy (i.e. explicit recognition of others' thoughts and emotion), and affective empathy (i.e. sharing another's perceived emotional state) and found that OT increased affective empathy, but not mentalizing (Geng et al., 2018; Hurlemann et al., 2010). However, Tomova et al. (2019) found OT enhanced mentalizing by sharpening self-other distinction during a perspective taking task.

Although there is limited evidence for a behavioral effect of OT on mentalizing, it is possible that OT affects neural regions associated with mentalizing even in the absence of behavioral changes. The mentalizing network includes the temporoparietal junction (TPJ)/posterior superior temporal sulcus, the medial prefrontal cortex (mPFC), the inferior frontal gyrus (IFG), the temporal poles, and the posterior cingulate cortex (PCC)/precuneus (Mar, 2011; Molenberghs, Johnson, Henry, & Mattingley, 2016; Schurz, Radua, Aichhorn, Richlan, & Perner, 2014; Van Overwalle & Baetens, 2009). To our knowledge, only three studies have used functional magnetic resonance imaging (fMRI) to examine the effect of OT on brain networks that underlie mentalizing in healthy participants. One study found no neural effects of OT on mentalizing using the Multifaceted Empathy Test (Dziobek et al., 2008) in a sample of men and women (*n* = 69) (Geng et al., 2018). However, the only region of the mentalizing network included as a region of interest (ROI) in this study was the mPFC. Another small study (*n* *=* 9) found some evidence for neural effects of OT on mentalizing using the RMET, but these results were not maintained after contrasting them with a gender identification control task (Pincus et al., 2010). Riem et al. (2014) also used the RMET and found OT increased activation in the insula, IFG superior temporal gyrus, and left paracingulate gyrus using ROI analyses in an all-female sample (*n* *=* 50). Considering that these three studies either performed ROI analysis that included very few mentalizing network regions, and/or employed the RMET, it remains unclear how OT affects neural activation specifically during mentalizing.

Compared to OT, even less research has examined the effects of AVP on mentalizing. In one study, AVP reduced mentalizing ability in the RMET (*n* = 39 males) (Uzefovsky et al., 2012), and an fMRI study (*n* = 39 males) on inferring emotions during social scenes (i.e. mentalizing) found that AVP increased activity in the PCC, lateral PFC, precentral gyrus, and left insula (Brunnlieb et al., 2013). These associations align with meta-analyses and reviews that have characterized a network of mentalizing neural correlates (Mar, 2011; Molenberghs et al., 2016; Schurz et al., 2014; Van Overwalle & Baetens, 2009).

In the present study, we recruited a large sample (*n* *=* 186) to examine the effects of OT or AVP compared to placebo on behavioral responses, neural activation, and functional connectivity when engaged in mentalizing. Participants completed the Why/How Task, which activates a network of mentalizing-related neural regions including the mPFC, lateral orbitofrontal cortex, PCC/precuneus, temporal poles, and the left TPJ (Spunt & Adolphs, 2014; Spunt & Lieberman, 2012b; Spunt, Satpute, & Lieberman, 2011). We also conducted several exploratory analyses including relevant moderators. Although evidence to date suggests that intranasal OT (and possibly AVP) administration may not affect mentalizing, the limitations of the RMET as an assessment of mentalizing point to the possibility that the use of a task that more robustly activates the mentalizing network may elucidate previously unknown effects. In addition, several studies have found effects of OT and AVP when completing tasks that engage social cognition, even if they are not specifically isolating the mentalizing process (Brunnlieb et al., 2016; Declerck, Boone, & Kiyonari, 2010, 2013; Feng et al., 2015; Gozzi, Dashow, Thurm, Swedo, & Zink, 2017; Rilling et al., 2012, 2014; Teed, Han, Rakic, Mark, & Krawczyk, 2019). We therefore hypothesized OT and AVP would modulate activation in, and/or connectivity between, neural regions in the mentalizing network.

## Methods

### Participants

All participants (*n* = 197) were recruited from the University of California, Los Angeles (UCLA) between January 2015 and July 2016 (see online Supplementary Information for exclusion criteria and online Supplementary Fig. S1 for CONSORT flow chart). The initial sample size collected for the OT (*n* = 75) and placebo (*n* = 89) groups provided 80% power to detect an effect size of 0.45 and 40% power to detect an effect size of 0.27. The initial sample size for AVP provided 80% power to detect an effect size of 0.57 and 40% power to detect an effect size of 0.35.^†^^1^ Five participants did not complete the Why/How Task due to lack of time, or technical issues. Further, participants were removed prior to analysis due to brain abnormalities (*n* = 2), brain alignment issues (*n* = 2), falling asleep in the scanner (*n* = 1), or being the pilot participant (*n* = 1). This resulted in 186 participants (ages 18–28, mean = 20.3, s.d. = 1.72, 119 female) who were randomly assigned in a double-blind procedure to receive OT (*n* = 71; 38 females), AVP (*n* = 31; 31 females), or placebo (*n* = 84; 50 females). Participants identified as Asian (41.4%), White (32.3%), Black or African American (2.2%), Native American or Alaska Native (0.5%), and ‘Other’ (23.7%). Across all participants, 27.4% identified as Hispanic or Latino. Participants who completed the study were paid $100. Informed consent was obtained from all participants as approved by the UCLA Institutional Review Board. The authors assert that all procedures contributing to this work comply with the ethical standards of the relevant national and institutional committees on human experimentation and with the Helsinki Declaration of 1975, as revised in 2008.

### Procedure

Participants completed two sessions (see online Supplementary Fig. S2 for study timeline). In the first session, participants completed a series of self-report questionnaires. The measures relevant to the present study are described below. The second session occurred 7–145 days later (mean = 45.37, s.d. = 26.41). Random assignment, dosage (i.e. 24 IU/ml for OT and 20 IU/ml for AVP), method of administration, and incubation period were conducted in the same manner as our previous research (Tabak et al., 2015, 2016, 2019; see online Supplemental Information for additional information). Following approximately 40 min of incubation, participants began preparation for the fMRI session. During fMRI scanning, participants performed other tasks (not analyzed for this paper) before the mentalizing task, which occurred approximately 90 min post-administration (as in Bartz et al., 2010).

### Why/How task

**Fig. 1.**
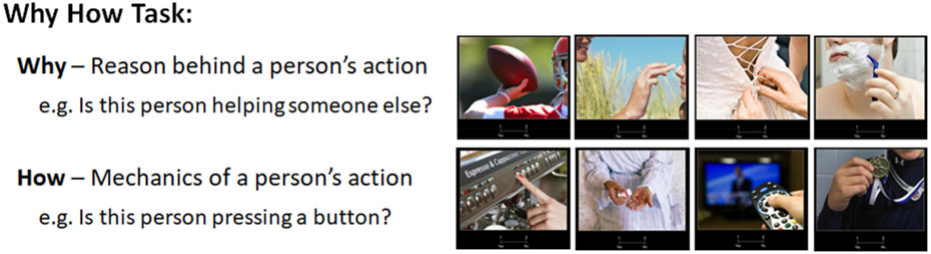
Description of Why/How Task. Following a fixation cross presented for an average of 9 s, each of the 16 experimental blocks began with a prompt question shown for 2.1 s followed by a blank screen lasting 0.15 s before presenting a sequence of eight trial images. Participants were given a max of 2.2 s to respond to each image, and a reminder prompt lasting 0.3 s was shown between each image. Each pre-block prompt began with ‘Is the person’ followed by a descriptive phrase specific to each question. This same phrase was then shown as a reminder between each trial. Recorded BOLD signal was analyzed in a variable epoch manner beginning from the onset of the first image to the offset of the final image of the block. The Why prompts described either intentions inferred from a person's bodily actions (e.g. ‘helping someone’) or emotional states inferred from facial expressions (e.g. ‘proud of themselves’). The How prompts described the physical mechanics of body actions (e.g. ‘lifting something’) or facial orientations (e.g. ‘opening their mouth’). The same set of images were used for both Why and How trials, and participants responded ‘yes’ or ‘no’ with their index or middle finger to indicate whether the person(s) in each image demonstrated that mental state or were performing the action stated in the prompt. On average, the task lasted approximately 4.9 min per person. See online Supplemental Information for additional prompt examples and task information.

The Why/How task (Spunt & Lieberman, 2012*a*, *b*; Spunt, Meyer, & Lieberman, 2015) reliably activates the mentalizing (Why > How) and mirror (How > Why) networks in the brain. We used a version that was nearly identical to study 3 in Spunt and Adolphs (2014) with minor adjustments to the trial structure timing (see Fig. 1 for additional information). Participants answered *why* (e.g. ‘Is the person helping someone’) someone is performing an action or *how* (e.g. ‘Is the person reaching’) someone is performing an action.

### Self-report measures

As shown in online Supplementary Table S1, participants completed several self-report measures for exploratory moderation analyses of OT and AVP effects.

### fMRI image acquisition

We collected data on a Prisma 3-T MR system at the UCLA Ahmanson-Lovelace Brain Mapping Center. We collected 148 functional volumes using a T2* weighted gradient echo-planar sequence with the following parameters: matrix size = 64 × 64, 3.1 × 3.1 × 3 mm voxels, repetition time (TR) = 2.0 s, echo time (TE) = 24 ms, flip angle (FA) = 90°, FOV = 1200 mm, bandwidth = 2605 Hz/Px, 20-channel head coil, and no acceleration. Volumes consisted of 36.3 mm slices with a distance factor 33%, and slice orientation tilt of 22.5% relative to the AC/PC plane. At the end of the scanning session, a high-resolution structural volume (MPRAGE) was collected with the sequence parameters: 1.1 × 1.1 × 1.2 mm voxels, TR = 2.3 s, TE = 2.95 ms, FA = 9°, distance factor 50%, and parallel imaging implementation mode GRAPPA with an acceleration factor of 2. The first 96 participants were run with scans going from posterior to anterior. Since dropout of signal can depend on the direction of the scans, for optimal coverage of the ventral PFC, we ran the last 90 participants with scans going from anterior to posterior. Contrasting blood oxygen level-dependent (BOLD) activation for participants whose images had posterior to anterior *v.* anterior to posterior encoding revealed a signal difference only in the inferior ventromedial prefrontal cortex (vmPFC) signal located behind the frontal sinuses. Thus, we did not expect the coding direction to affect treatment group contrasts elsewhere in the brain.

### Statistical analysis

#### Behavioral analysis

We examined the effects of either neuropeptide on reaction time and accuracy for the Why/How task using two-sided *t* tests and report the 95% confidence intervals (CIs) as well as estimated effect sizes calculated by Cohen's *d*. Accuracy was based on Spunt and Adolphs's (2014) coding of each image. We chose to focus specifically on OT *v.* placebo or AVP *v.* placebo because we did not have *a priori* hypotheses about OT *v.* AVP.

#### fMRI analysis

*General linear model.* The imaging data were analyzed in SPM12 (Wellcome Department of Imaging Neuroscience, Institute of Neurology, London, UK). Functional volumes were motion and distortion corrected, normalized to a standardized (MNI) template using the DARTEL toolbox (including resampling to 2 mm isotropic voxels), spatially smoothed with a Gaussian kernel (8 mm FWHM) and high-pass filtered (128 s cut-off). Analyses were run on the whole brain as well as on ROIs defined in Spunt and Adolphs (2014). Drug condition (OT, AVP, or placebo) was added as a covariate in the second-level analysis.

*GLM Fast Flex 2.* To ensure our ability to detect effects in areas prone to drop out, we used GLM Fast Flex 2 (Harvard University, Boston, USA) to conduct whole-brain, voxel-level analysis. This package has the benefit of calculating voxels in which some participants might be missing data. GLM Fast Flex 2 was also used to conduct exploratory moderation analyses involving self-report measures. These self-reports were included as moderators in the second-level analysis.

*Functional connectivity.* We analyzed the functional connectivity during each task block and during rest using the CONN toolbox (www.nitrc.org/projects/conn; version 17.f). The data were processed with band-pass filtering between 0.01 and 0.1 Hz and the default component-based CompCor method for reducing physiological and other sources of noise (Whitfield-Gabrieli & Nieto-Castanon, 2012).

*ROI analyses.* Twenty ROIs (10 from the mentalizing network, 10 from the mirror network) were collected from clusters found to be significant at *p* < 10^−6^ uncorrected from a previously validated version of the Why/How contrast by Spunt and Adolphs (2014). We extracted the *β* values within each ROI for each participant. Each ROI was tested separately in contrasts for each of the OT or AVP *v.* placebo contrasts. Additionally, to increase signal to noise, *a priori* clusters associated with the mentalizing or mirror networks were grouped and contrasted for group effects as two network ROIs. For the functional connectivity analysis, bivariate correlations from the CONN toolbox were examined between each of the 10 ROIs within the mentalizing and mirror networks respectively. Mentalizing ROIs included the PCC/precuneus, mPFC, vmPFC, left posterior middle frontal gyrus, left and right TPJ, left and right STS, and left and right ventral lateral PFC. Mirror network ROIs included bilateral intraparietal lobule, bilateral posterior premotor cortex, bilateral posterior IFG, bilateral posterior middle temporal gyrus, and bilateral superior parietal lobule.

All statistical tests were performed in MATLAB (MathWorks, Natick, MA, USA) and report *p* values, CIs, effect size, Bayes factors (BF), and equivalence tests. BFs were calculated as ratios of the marginal likelihoods of the data under the alternative and null hypothesis given a prior odds distribution of *r* = 0.707, the default prior distribution in the BayesFactor package in R from which we extracted code (Morey & Roulder, 2015). The lack of support in the literature for hypothesizing specific behavioral results in this task, or of commonly reported effect sizes in BOLD ROI analyses, limited us to the use of a default prior distribution which favors the null hypothesis in cases where there is a true small effect (Rouder, Speckman, Sun, Morey, & Iverson, 2009). Therefore, the BF results should be viewed with a degree of skepticism.

## Results

### Oxytocin and vasopressin effects on accuracy and reaction time

**Fig. 2.**
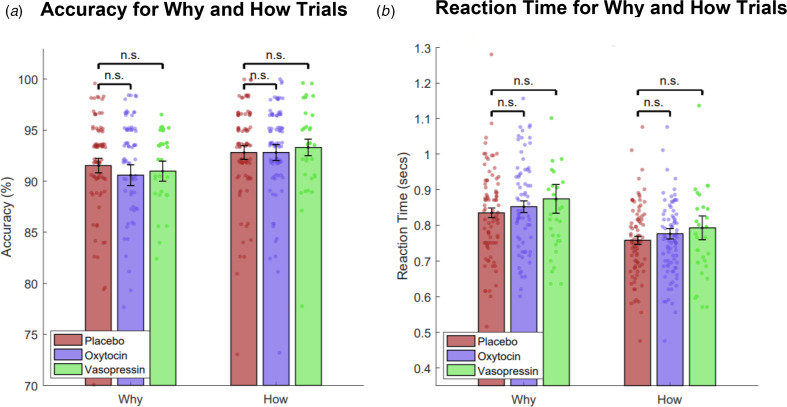
Differences between OT, AVP, and placebo on accuracy and reaction time. (a) Accuracy for Why and How trials, (b) reaction time for Why and How trials. No significant behavioral differences in accuracy or reaction time were observed for OT and AVP *v.* placebo in either the Why or How trials. Error bars represent standard error. *p* < 0.001***, *p* < 0.01**, *p* < 0.05*, *p* < 0.10♱, n.s. = not significant.

We first examined the effect of either neuropeptide on accuracy and reaction time during Why trials (mentalizing) and How trials (action understanding). No significant differences between treatment groups were found in accuracy (Fig. 2*a*) or reaction time (Fig. 2*b*) for either mentalizing or action understanding, when comparing either OT or AVP to placebo (see online Supplemental results).

### Oxytocin and vasopressin effects on neural activation

**Fig. 3.**
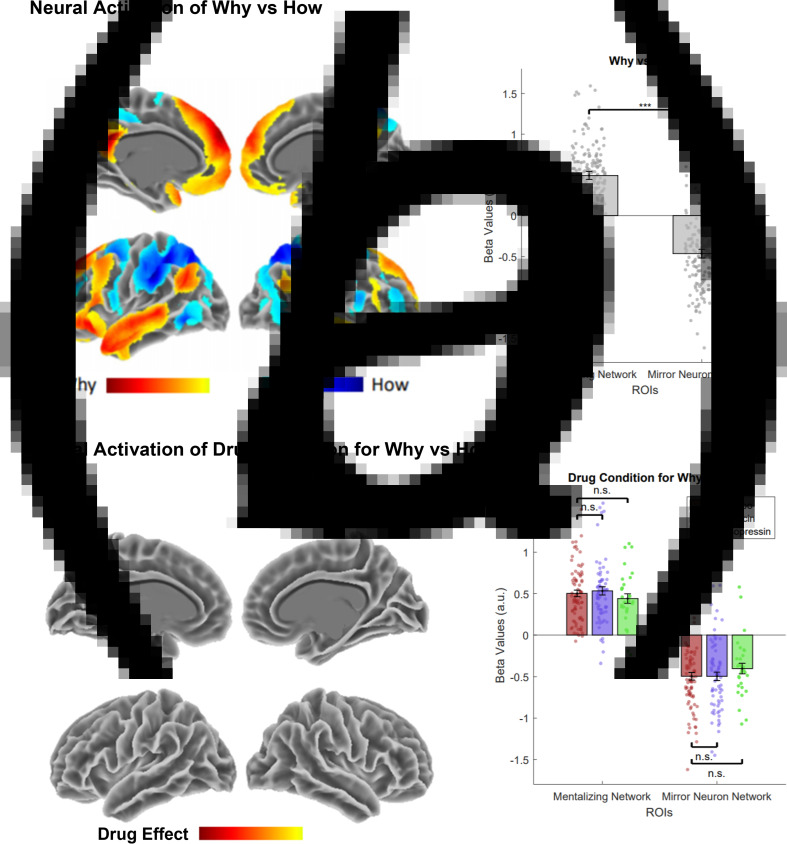
Differences in neural activity found via *t* tests contrasting the effects of either OT or AVP *v*. placebo. (a) Neural activation of Why *v*. How, (b) neural activation of drug condition for Why *v*. How. (a) The contrast of Why *v*. How activation collapsed across all conditions shows robust activation in the mentalizing network ROI for Why trials and mirror network ROI for How trials. Warmer colors correspond to greater activation during Why trials and cooler colors to greater activation during How trials. (b) The graph on the right shows there is no significant difference in neural activation for either OT or AVP *v*. placebo (*p* > 0.05) in the mentalizing network ROI for Why trials and mirror network ROI for How trials. The maps on the left show no significant activation (*p* < 0.001 uncorrected) for either drug condition. Error bars represent standard error. *p* < 0.001***, *p* < 0.01**, *p* < 0.05*, *p* < 0.10♱, n.s. = not significant.

We then examined the effects of either neuropeptide on brain activation during the Why/How task as measured by BOLD response. We first analyzed the main effect of the Why *v.* How contrast across all participants (including OT, AVP, and placebo groups). Replicating prior work, we found robust activation of the mentalizing network ROI for the Why compared to How trials in the placebo group [*t*_83_ *=* 12.621, *p* *<* 1 × 10^−10^, 95% CI (0.42–0.58), Cohen's *d* *=* 1.377] and when collapsing across all drug conditions [*t*_185_ = 18.1, *p* < 1 × 10^−10^, 95% CI (0.45–0.56), Cohen's *d* = 1.33] (Fig. 3a). We also found robust activation of the mirror network ROI for How compared to Why trials in the placebo group [*t*_83_ = 10.82, *p* < 1 × 10^−10^, 95% CI (0.59–0.40), Cohen's *d* = 1.181] and when collapsing across all drug conditions [*t*_185_ = 15.893, *p* *<* 1 × 10^−10^, 95% CI (0.54–0.42), Cohen's *d* *=* 1.165] (Fig. 3*a*).

We next examined the effects of OT or AVP *v.* placebo on the Why *v.* How trials. When conducting whole-brain analyses, no effect was found for OT or AVP compared to placebo for the whole-brain contrast at a liberal threshold of *p* < 0.001 uncorrected at either the voxel or cluster level (Fig. 3*b*). Next, when comparing *β* values for the mentalizing *v.* mirror networks, we found no effect of Why *v.* How in the mentalizing network for OT *v.* placebo or AVP *v.* placebo, nor did we find any effect for the reverse How *v.* Why contrast in the mirror network for OT *v.* placebo or AVP *v.* placebo (Fig. 3*b*). Finally, when examining each of the ROIs within the networks, we also found no effects of OT *v.* placebo (Table 1) or AVP *v.* placebo (Table 2) in any ROI of either the mentalizing or mirror networks (see online Supplementary Fig. S3 for equivalence test results). AVP did demonstrate a positive moderate effect on each of the left and right premotor cortices of the mirror network compared to placebo when How was compared to Why trials (Left: BF10 = 4.92; Right: BF10 = 7.39), but this did not maintain significance following multiple test correction using the False Discovery Rate (FDR; Benjamini & Hochberg, 1995) in *t* tests [Left: *t*_56_ *=* 2.700, *p*_adj_ = 0.09, 95% CI (0.064–0.432), Cohen's *d* *=* 0.642; Right: *t*_65_ *=* 2.872, *p*_adj_ = 0.09, 95% CI (0.075–0.418), Cohen's d *=* 0.652].

**Table 1.** Neural region of interest signal change differences between oxytocin and placebo groups as tested via both *t* tests and Bayes factors

ROI name	Null hypothesis significance testing							Bayes factor	
	df	*t*-value	*p* value uncorrected	*p* value FDR corrected	Lower 95% CI	Upper 95% CI	Cohen's *d*	BF01	BF10
Why network	139.6	0.482	0.630	0.990	−0.096	0.157	0.079	5.17	0.193
PCC	148.8	0.304	0.761	0.980	−0.108	0.147	0.049	5.52	0.181
mPFC	150.1	0.038	0.970	0.980	−0.114	0.119	0.006	5.76	0.174
vmPFC	145.3	0.062	0.951	0.980	−0.095	0.101	0.010	5.75	0.174
LpMFG	151.0	0.315	0.753	0.980	−0.118	0.163	0.051	5.50	0.182
LTPJ	138.4	0.234	0.815	0.980	−0.150	0.190	0.038	5.62	0.178
RTPJ	151.4	0.036	0.971	0.980	−0.201	0.209	0.006	5.76	0.174
LSTS	150.0	1.074	0.285	0.980	−0.031	0.105	0.173	3.39	0.295
RSTS	142.6	0.050	0.960	0.980	−0.062	0.066	0.008	5.75	0.174
LvlPFC	147.5	0.421	0.675	0.980	−0.071	0.110	0.068	5.31	0.188
RvlPFC	139.7	0.026	0.980	0.980	−0.084	0.086	0.004	5.76	0.174
How network	147.7	0.013	0.990	0.990	−0.134	0.136	0.002	5.76	0.174
LIPL	144.4	0.346	0.730	0.980	−0.105	0.149	0.056	5.45	0.183
RIPL	143.4	0.996	0.321	0.980	−0.060	0.181	0.162	3.65	0.274
LpPMC	134.5	0.475	0.635	0.980	−0.100	0.164	0.078	5.19	0.193
RpPMC	142.2	0.968	0.335	0.980	−0.069	0.202	0.157	3.74	0.267
LpIFG	131.1	0.148	0.883	0.980	−0.099	0.115	0.024	5.70	0.175
RpIFG	147.2	0.196	0.845	0.980	−0.084	0.102	0.032	5.66	0.177
LpMTG	142.0	0.271	0.787	0.980	−0.137	0.181	0.044	5.57	0.180
RpMTG	146.9	0.673	0.502	0.980	−0.117	0.238	0.109	4.68	0.214
LSPL	148.2	0.325	0.745	0.980	−0.162	0.226	0.052	5.49	0.182
RSPL	148.7	0.768	0.444	0.980	−0.121	0.274	0.124	4.39	0.228
Amygdala	150.9	0.359	0.720	0.990	−0.073	0.105	0.056	5.43	0.184

**Table 2.** Neural region of interest signal change differences between vasopressin and placebo groups as tested via both *t* tests and Bayes factors

ROI name	Null hypothesis significance testing							Bayes factor	
	df	*t*-value	*p* value uncorrected	*p* value FDR corrected	Lower 95% CI	Upper 95% CI	Cohen's *d*	BF01	BF10
Why network	57.1	0.459	0.648	0.648	−0.109	0.173	0.108	3.85	0.259
PCC	59.0	1.019	0.312	0.481	−0.101	0.311	0.238	2.70	0.371
mPFC	55.8	0.253	0.801	0.890	−0.168	0.217	0.060	4.11	0.243
vmPFC	58.8	0.458	0.649	0.865	−0.122	0.194	0.107	3.86	0.259
LpMFG	61.0	0.499	0.620	0.865	−0.149	0.248	0.116	3.79	0.264
LTPJ	53.5	1.047	0.300	0.481	−0.115	0.367	0.252	2.63	0.381
RTPJ	59.9	1.019	0.312	0.481	−0.155	0.478	0.237	2.70	0.371
LSTS	65.4	0.280	0.780	0.890	−0.090	0.120	0.064	4.08	0.245
RSTS	60.5	0.129	0.898	0.945	−0.083	0.094	0.030	4.19	0.238
LvlPFC	66.3	0.004	0.997	0.997	−0.132	0.132	0.001	4.22	0.237
RvlPFC	56.5	0.353	0.725	0.890	−0.104	0.149	0.084	4.00	0.250
How network	60.4	0.971	0.335	0.648	−0.081	0.234	0.226	2.81	0.356
LIPL	57.4	2.003	0.050	0.184	0.000	0.365	0.472	0.77	1.303
RIPL	50.8	1.698	0.096	0.217	−0.031	0.369	0.415	1.23	0.811
LpPMC	55.7	2.700	**0**.**009**	0.092	0.064	0.432	0.642	0.20	**4**.**918**
RpPMC	65.1	2.872	**0**.**005**	0.092	0.075	0.418	0.652	0.14	**7**.**393**
LpIFG	62.2	1.281	0.205	0.410	−0.046	0.209	0.295	2.08	0.480
RpIFG	58.3	1.758	0.084	0.217	−0.015	0.234	0.413	1.13	0.887
LpMTG	59.5	1.683	0.098	0.217	−0.038	0.445	0.393	1.26	0.796
RpMTG	57.5	1.956	0.055	0.184	−0.006	0.510	0.461	0.83	1.207
LSPL	57.4	2.342	**0**.**023**	0.151	0.047	0.596	0.552	0.42	2.388
RSPL	53.4	2.105	**0**.**040**	0.184	0.015	0.597	0.507	0.65	1.545
Amygdala	61.8	0.613	0.542	0.648	−0.102	0.193	0.141	3.59	0.279

*Note.* Bold = *p* < 0.05 and/or BF10>3.

### Oxytocin and vasopressin effects on functional connectivity

**Fig. 4.**
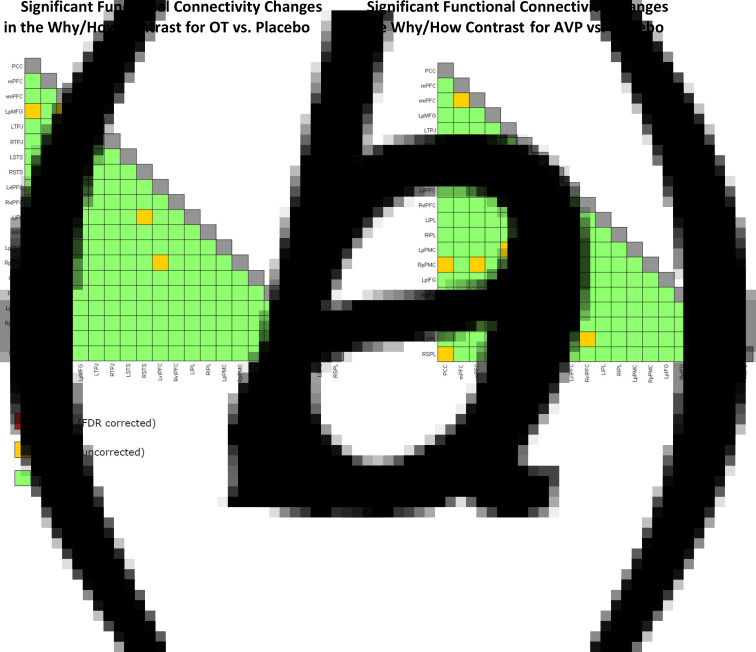
Twenty ROIs chosen from Spunt and Adolphs (2014). (a) Significant functional connectivity changes in the Why/How contrast for OT *v*. placebo. (b) Significant functional connectivity changes in the Why/How contrast tasks for AVP *v*. placebo. *Note.* The ROIs used are very similar to the significant activations shown in Fig. 3*a* (Why *v*. How contrast). (a) No significant difference in functional connectivity during Why compared to How trials for OT *v*. placebo. (b) No significant difference in functional connectivity during Why compared to How trials for AVP (only females) *v*. placebo (only females). Green squares for *p* > 0.05, yellow squares for *p* < 0.05 uncorrected for multiple comparisons, red squares for *p* < 0.05 FDR corrected, and gray squares for correlations of ROI with itself. PCC, posterior cingulate cortex/precuneus; mPFC, medial prefrontal cortex, vmPFC, ventromedial prefrontal cortex; LpMFG, left posterior middle frontal gyrus; LTPJ, left temporoparietal junction; RTPJ, right temporoparietal junction; RSTS, right superior temporal sulcus; LSTS, left superior temporal sulcus; LvlPFC, left ventral lateral prefrontal cortex; RvlPFC, right ventral lateral prefrontal cortex; LIPL, left intraparietal lobule; RIPL, right intraparietal lobule; LpPMC, left posterior premotor cortex; RpPMC, right posterior premotor cortex; LpIFG, left posterior inferior frontal gyrus; RpIFG, right posterior inferior frontal gyrus; LpMTG, left posterior middle temporal gyrus; RpMTG, right posterior middle temporal gyrus; LSPL, left superior parietal lobule; RSPL, right superior parietal lobule.

We also examined functional connectivity while the participants were performing the task. First, we measured how changes in connectivity for the Why *v.* How contrast differed for each of the OT and AVP conditions compared to the placebo group. The connectivity between 20 ROIs provided 190 comparisons for both drugs. After FDR correction, we found no significant difference in connectivity during Why compared to How trials for either OT or AVP *v.* placebo between any of the 20 ROIs (Fig. 4*a* and *b*; online Supplementary Fig. S3).

### Exploratory analyses of gender

We further examined potential gender interaction effects with OT *v.* placebo on accuracy and reaction time for Why/Why and How/How trials and found no effects (see online Supplementary Table S2 and online Supplementary Figs S3 and S4). We also found no significant differences in whole-brain and ROI analyses, nor functional connectivity in men or women (online Supplementary Figs S3, S5, and S6).

### Exploratory moderation analyses

To explore potentially relevant moderators of OT or AVP effects, we separately averaged the activity observed within each of the mentalizing and mirror networks from Spunt and Adolphs (2014). To aid interpretation, we *z*-scored the neural data and each moderator across participants and included each moderator individually in a regression as an interaction with the dummy-coded drug conditions. We examined comparisons using both a liberal nominal significance (*p* < 0.01 uncorrected) and the FDR correction (*q* = 0.05). No interaction effects were observed for the mentalizing network. We found an interaction only for the Autism Spectrum Quotient × OT *v.* placebo in the mirror network [*b* = −0.494, *t*(180) = −2.99, *p* = 0.0032] that showed nominal significance (*p* < 0.01 uncorrected) (online Supplementary Fig. S7), but this did not survive multiple test correction (FDR corrected *p* = 0.14).

## Discussion

In this large fMRI study, we found no effects of OT or AVP *v.* placebo on either neural activation or functional connectivity when contrasting inferences of Why *v.* How a behavior was performed. We also observed no behavioral differences between OT or AVP *v.* placebo for task accuracy or response times. The absence of effects is noteworthy considering that highly significant differences in neural activation between Why/How conditions were found when collapsing across drug and placebo groups. Thus, these null findings do not appear to result from a failure of the Why/How task to elicit mentalizing or mirror networks. Rather, these findings represent a lack of evidence that either neuropeptide influences mentalizing at the behavioral or neural level.

Meta-analyses have found evidence of beneficial effects of OT on theory of mind in studies of people with neurodevelopmental disorders (Bürkner, Williams, Simmons, & Woolley, 2017; Keech, Crowe, & Hocking, 2018), and preliminary evidence suggests that AVP administration may improve social ability in children with autism spectrum disorders (Parker et al., 2019). In line with these findings, studies with healthy samples have found OT enhanced performance on social tasks among individuals with lower levels of self-reported social cognitive ability (Bartz et al., 2010, 2019; Feeser et al., 2015; Luminet et al., 2011; Radke & de Bruijn, 2015). Given these prior findings, we examined several self-report measures of social cognitive ability, as well as other relevant potential moderators, but found no evidence to support moderation effects following correction for multiple testing.

The lack of results for OT or AVP suggests that these neuropeptides may not influence mentalizing in healthy individuals. This is consistent with the results from Geng et al. (2018) who found null results for mentalizing using the Multifaceted Empathy Task (Dziobek et al., 2008) which features contextually and visually rich stimuli similar to those in the Why/How task presented here. We also extended the findings of Geng et al. (2018) to regions beyond the mPFC, amygdala and anterior insula by conducting a whole-brain analysis as well as targeted analyses for several regions in the mentalizing and mirror networks.

It must be noted that there is still skepticism about the use of intranasal OT and AVP administration due to methodological issues, and in particular, the unclear pharmacokinetics (Leng & Ludwig, 2016; but see Quintana et al., 2021 for recent advances). Furthermore, failed replications (Declerck, Boone, Pauwels, Vogt, & Fehr, 2020; Nave, Camerer, & McCullough, 2015), a publication bias against null results (Lane, Luminet, Nave, & Mikolajczak, 2016; Tabak et al., 2019), and issues of statistical power have promoted uncertainty regarding all but the most consistent findings in the OT literature (Mierop et al., 2020; Walum, Waldman, & Young, 2016). To date, the effects of OT appear more robust for basic processes such as emotion recognition (Leppanen et al., 2017) and attention orientation toward social cues (Eckstein et al., 2019; Guastella, Mitchell, & Dadds, 2008; Hubble et al., 2017). While these processes are certainly relevant for mentalizing, they are not one and the same as engaging in mentalizing. Thus, it is possible that OT plays a greater role in the basic mechanisms that are foundational for social cognitive ability (Mitchell & Phillips, 2015).

The present results may also differ from previous studies using the RMET because it is possible that OT may more strongly affect emotion recognition than mentalizing processes. Although mentalizing and emotion recognition networks show overlap in the mPFC (Lieberman, Straccia, Meyer, Du, & Tan, 2019) and the IFG (Hooker, Verosky, Germine, Knight, & D'Esposito, 2008), emotion recognition tasks are typically more associated with activity in limbic and paralimbic regions such as the amygdala, anterior insula, and parahippocampal gyrus, as well as the fusiform face area and other visual processing areas (Dricu & Frühholz, 2016; Fusar-Poli et al., 2009; Sabatinelli et al., 2011). Based on meta-analytic evidence showing that OT tends to affect limbic regions, particularly the amygdala and insula (Grace, Rossell, Heinrichs, Kordsachia, & Labuschagne, 2018; Zink & Meyer-Lindenberg, 2012), emotion recognition tasks that engage these regions may be more susceptible to OT's influence, whereas mentalizing tasks that engage broader cortical networks may be less affected.

Another possibility for the present findings is that the main effects of the Why/How task are too strong to see drug effects. Indeed, one of the primary strengths of the Why/How task compared to other mentalizing paradigms, such as the RMET, is the tight control it offers in parsing neural effects related to mental state inference *v.* inferences of the physical mechanics of behaviors signaling those mental states (Spunt, Falk, & Lieberman, 2010). However, the near ceiling behavioral accuracy of the Why/How task (i.e. >90%) is also a limiting factor in our analysis, particularly in relation to identifying behavioral effects of OT or AVP. As such, future studies examining the behavioral effects of OT or AVP on mentalizing would benefit from the inclusion of a similarly well-validated mentalizing task that is more challenging for healthy individuals.

The present study has a number of strengths including the use of one of the most well-validated fMRI tasks for reliably dissociating the mentalizing and mirror networks, indicating the rare ability amongst fMRI tasks of distinguishing mental state representations from perceptual and motor representations. We also demonstrate null results of both main effects and interaction effects related to measures previously observed to moderate the effects of OT or AVP on social cognitive processes.

Since the majority of human studies examining the effects of OT and AVP has relied on all male samples, our majority female sample can be viewed as a strength and represents an important contribution to the literature (Quintana et al., 2021). At the same time, the inclusion of a majority female sample prevents us from knowing the extent to which these results would be confirmed in a sample with an equal number of male participants. In addition, recruiting only healthy participants does not allow us to know whether the present results may generalize to those with psychiatric disorders. The present results also relied on a single, conventional dose of OT and AVP, and it is unclear whether results would remain consistent with a lower or higher dose of either neuropeptide.

In addition, our results only relate to the effects of OT or AVP approximately 90 min post-administration. The current consensus for the optimal amount of time for incubation is approximately 35–50 min before beginning the task. Therefore, our results may not represent the peak increase in OT concentrations (Quintana et al., 2021). Nonetheless, studies have used similar incubation periods and found effects of OT (Bartz et al., 2010). There is also evidence that OT remains increased in cerebrospinal fluid and blood plasma for 75–85 min and beyond following OT administration (Spengler et al., 2017; Striepens et al., 2013), and intranasal OT has produced sustained changes in resting regional cerebral blood flow in numerous regions implicated in socioemotional processing through 78 min post-administration (Paloyelis et al., 2016). Future studies are needed to examine if the null results found in the present study extend to shorter or longer incubation periods, as well as larger or repeated doses.

Another limitation is that our between-subjects design limited overall power (Quintana et al., 2021). Future studies may consider a within-subjects design to increase power. In addition, although we observed general agreement regarding our null findings across different types of analyses, inconsistencies in the OT administration literature prevented us from using a better-informed prior distribution. Thus, we were limited to using a default prior distribution. The smaller a potential true effect is, the more the default prior distribution can be considered to overweigh larger effect sizes. Small effects are then farther from the average distribution effect and less distinguishable from zero, introducing bias toward the null hypothesis (Rouder et al., 2009). Thus, since the effects of OT and AVP in the Why/How task may be small, and we cannot know how small, the interpretation of BF results should be qualified accordingly.

Last, it is important to note that in our preregistration in 2015, the Why/How task was overlooked and not listed because it was initially intended as a localizer for use in a separate task in the same broader study (a task described as ‘learning for teaching’). In addition, unfortunately, for logistical purposes, the larger study was separated into three different preregistrations based on different funding mechanisms (NCT02393443; NCT02393456; NCT02394054). Information on the overall study design and the other tasks, including an empathy task, a deception detection task, and a task involving viewing images of participants' attachment figures, were listed in the original preregistrations. Null results that are not preregistered, such as those in the present study, may be subject to biases such as reverse *p*-hacking (Chuard, Vrtílek, Head, & Jennions, 2019). Nevertheless, the results ran counter to our hypotheses and extensive analyses were completed to allow OT and AVP effects to present themselves.

In sum, in a majority female non-clinical sample, we found no effects of either OT or AVP administration compared to placebo on behavioral responses, neural activation, or functional connectivity related to mentalizing, a social cognitive process that is impaired in several clinical populations. Furthermore, of the relevant moderators we explored, no associations survived after correction for multiple comparisons. These results, based on the well-validated Why/How task, support previous research showing a lack of an association between OT and several social processes (Tabak et al., 2019), including mentalizing, at the behavioral level (Radke & de Bruijn, 2015). Moreover, by examining whole-brain and network effects, we add to prior work that has shown a lack of an association at the neural ROI level.

## Footnotes

† The notes appear after the main text.

## Notes

1 Based on time and resources, we had the opportunity to include AVP administration for approximately 30 individuals. Given that we could only include this number, we chose to include only female participants in this condition because the vast majority of AVP administration studies have only included male participants (Brunnlieb et al., 2013; Gozzi et al., 2017; Thijssen et al., 2018).

## Supplementary material

For supplementary material accompanying this paper visit https://doi.org/10.1017/S0033291721004104. click here to view supplementary material
