# Supplementary material for: Null results of oxytocin and vasopressin administration on mentalizing in a large fMRI sample: evidence from a randomized controlled trial
Source: Psychol Med. 2021 Oct 15;53(6):2285–95. doi: 10.1017/S0033291721004104 (PMC10123837; doi:10.1017/S0033291721004104)
Supplement: Supplementary file 1 [file S0033291721004104sup001.docx]

**Supplementary Information**

**Exclusion criteria**

Exclusion criteria included being less than 18 years old or older than 30 years of age, current cold symptoms, restricted fluid intake for any reason, present or history of medical illness, present psychiatric diagnosis, present use of medications (e.g., SSRIs), breastfeeding, pregnancy, metal implants, smoking >15 cigarettes per day, drug use, and pregnancy. Participants were asked to refrain from using alcohol or medication for 24 hours, caffeine for 4 hours, and food or drinks (except water) for 2 hours preceding the experiment.

**Procedure**

During the drug administration session, participants first completed questionnaires including measures assessing state anxiety and positive/negative affect (see Supplementary Measures), and also provided a urine sample for drug and pregnancy testing. Nurses then checked the temperature, heart rate, and blood pressure of each participant to ensure that they were in the accepted limits: elevated blood pressure (>135/90), low blood pressure (<85/55), or body temperature (>100.1 Fahrenheit). If any of their vital signs were outside of the accepted range, participants rested for 10-15 minutes, and measurements were repeated until readings were within acceptable limits.

Prior to each session involving drug administration, a third-party research coordinator used an online random number generator ([www.random.org](http://www.random.org)) to randomly assign participants to receive either OT, AVP, or the placebo condition (blocked on gender) and communicated this information to the UCLA pharmacy. Participants then received one of the study drugs or placebo using a randomized, double-blind, placebo-controlled, between-subjects procedure. Substances were delivered using sterile 6 ml amber glass bottles with metered nasal pumps from Advantage Pharmaceuticals, Inc. Participants received instructions on how to use the nasal sprays and were then instructed to deliver one spray per nostril in an alternating fashion when prompted (every 30 seconds).

OT (Syntocinin) was provided by Novartis Pharmaceuticals, Switzerland. OT (24 IU/ml) was transferred into the bottles with attached intranasal applicators (1 puff=0.1ml). Participants self-administered 5 puffs per nostril (2.4 IU/puff) for a total dose of 24 IU. AVP was provided by American Regent Laboratories, Shirley, NY, USA. The pharmacist transferred AVP (20 IU/ml) into the bottles with attached intranasal applicators. Participants self-administered 5 puffs per nostril (2 IU/puff) for a total dose of 20 IU. Placebo (used previously by Tabak et al., 2016) consisted of 2 mls glycerine and 3 mls purified water (methylparaben and propylparaben mixed according to purified water formula) for a total of 5 ml. This was filtered with a 5 mu filter and transferred to the bottles with attached intranasal applicators.

As in previous research (Tabak et al., 2015, 2016), participants waited approximately 40 minutes following completion of administration before beginning the tasks. During this time, the experimenter provided instructions for the tasks that the participants would be completing in the scanner. Following approximately 10 minutes of instructions, participants were asked to sit quietly and read from a stack of outdated magazines (e.g., Newsweek). After the incubation period, participants once again completed measures of state anxiety and positive/negative affect (see Supplementary Measures). No adverse effects were reported following drug administration. Following the 40-minute incubation period, participants prepared for the fMRI session, which included placing MRI-safe pads on either side of participants’ heads to reduce head motion.

**Additional information on Why/How Task**

The Why questions require mentalizing about other’s actions, while the How questions require action representation or mirror neuron activity. Each experimental block started with either a Why or How prompt question followed by eight images in a row. The Why prompts described either intentions inferred from a person’s bodily actions (e.g., “competing against others,” “concerned with their health,” “helping someone,” and “protecting themselves”) or emotional states inferred from facial expressions (e.g., “admiring someone,” “expressing self-doubt,” “in an argument,” and “proud of themselves”). The How prompts described the physical mechanics of body actions (e.g., “lifting something,” “pressing a button,” “reaching for something,” and “using both hands”) or facial orientations (e.g., “smiling,” “looking at the camera,” “looking to their side,” and “opening their mouth”). The start of the next block began at a predetermined jitter time of around nine seconds. Participants were given one of four possible orders and timing sequences previously determined in Spunt and Adolphs (Spunt & Adolphs, 2014) to provide the best BOLD signal-to-noise ratio.

Importantly, the Why/How Task uses different instructional prompts for the same set of photographs. This helps control for possible lower-level visual confounds enabling direct contrast of identification of the intentions of depicted actions and meaning of emotionally expressive scenes (Why) against identification of the procedural mechanics that produce the action or emotional expression (How). Critically, this allows for isolation of neural activation related to the mentalizing network from that of the mirror network that associates the observation of an action with a motor representation (Iacoboni, 2009; Niedenthal, Mermillod, Maringer, & Hess, 2010) and allows for mimicry of actions and emotional expressions even in the absence of an explicit representation of the mental state producing the action or expression (Preston & de Waal, 2002).

**Rationale for choosing self-report measures for exploratory moderation analyses**

Based on studies showing interaction effects of OT on social cognitive processes (Bartz, Nitschke, Krol, & Tellier, 2019; Feeser et al., 2015), we conducted several exploratory analyses including relevant moderators. We analyzed these effects using a whole-brain approach as well as a more focused contrast of ROIs comparing mentalizing with the mirror networks for the Why versus How conditions. We hypothesized that OT and AVP would modulate activation in, and connectivity between, neural regions in the mentalizing network. In particular, we hypothesized that OT and AVP would increase activation in the MPFC and PCC, two regions previously observed to be affected by these neuropeptides in relevant social cognition tasks (Brunnlieb, Münte, Tempelmann, & Heldmann, 2013; Chen, Gautam, Haroon, & Rilling, 2017; Feng et al., 2015; Geng et al., 2018; Teed, Han, Rakic, Mark, & Krawczyk, 2019; Zhao et al., 2016).

Bartz and colleagues (Bartz et al., 2019, 2010) found a moderated effect of OT on empathic accuracy among individuals with greater social cognitive impairment based on scores from the Autism Spectrum Quotient (AQ; Baron-Cohen et al., 2001). Similarly, Feeser and colleagues (Feeser et al., 2015) found that OT increased mentalizing ability among individuals with lower levels of dispositional empathy as measured by the Empathy Quotient (EQ; Baron-Cohen and Wheelwright, 2004), and Radke and de Bruijn (Radke & de Bruijn, 2015) found results when examining the moderating role of the empathic concern subscale of the Interpersonal Reactivity Index (IRI; Davis, 1983). Luminet and colleagues (Luminet, Grynberg, Ruzette, & Mikolajczak, 2011) also found increased mentalizing ability following OT administration among individuals with higher levels of alexithymia. Most recently, a study by Tomova, Heinrichs, and Lamm (Tomova, Heinrichs, & Lamm, 2019) found that OT increased visual perspective taking when engaged in a behavioral task suggesting that individual differences in perspective taking could be a potential moderator of OT’s effects.

The moderating role of personality traits including extraversion and agreeableness have also been associated with altered effects of OT on social processes including prosocial behavior (Human, Thorson, & Mendes, 2016) and social reward (Groppe et al., 2013). Riem and colleagues (2014) found moderated effects of OT on mentalizing among individuals who experienced higher levels of maternal love withdrawal which led us to examine retrospective perceptions of mothers and fathers during childhood through the Parental Bonding Instrument (Parker, Tupling, & Brown, 1979). Relatedly, participants who experienced greater early life adversity showed increased emotion recognition ability following OT (Schwaiger, Heinrichs, & Kumsta, 2019), which led us to include the Risky Families Questionnaire (Taylor, Lerner, Sage, Lehman, & Seeman, 2004). Last, based on previous evidence that OT decreased social working memory accuracy in individuals with higher levels of social anxiety (Tabak et al., 2016), we included a composite measure of social anxiety as we have in our previous research. Thus, we examined the following moderators: AQ, EQ, alexithymia, extraversion, agreeableness, empathic concern, perspective taking, perceptions of early maternal and paternal care, early life adversity, and social anxiety.

**Measures**

***Pre-post administration questionnaires***

To measure non-specific effects of OT or AVP, we administered several questionnaires before neuropeptide/placebo administration (i.e., pre-administration), and again after the incubation period before participants entered the scanner (i.e., post-administration).

*State Anxiety.* We first measured state anxiety using the state version of the State Trait Anxiety Inventory (STAI; Spielberger et al., 1983). Items on the STAI were rated using a 4-point Likert-type scale (1=not at all; 4=very much). A mean composite was created for the STAI pre-administration (α=.89) and post-administration (α=.88). A change score was then computed (pre-administration - post-administration) to examine differences in state anxiety before and after drug-administration.

*State Positive and Negative Affect.* We also measured positive and negative affect using the 10-item PANAS (Thompson, 2007). Items on the PANAS were rated using a 5-point Likert-type scale (1=not at all; 5=extremely). Mean composites were created to represent positive affect (pre-administration α=.74; post-administration α=.80) and negative affect (pre-administration α=.58; post-administration α=.55). Change scores (pre-administration - post-administration) were then computed to examine differences in positive and negative affect before and after drug-administration.

**fMRI Image Processing**

For quality control purposes, we compared the default CompCor noise reduction method in the CONN toolbox to an alternative method of explicitly controlling for mean signal, white matter signal, ventricles signal, head movement, and first derivatives of head movement. Both methods provided similar results.

**Results**

***Accuracy***

We first examined the effect of either neuropeptide on accuracy during Why trials (e.g., “Is the person helping someone”) and How trials (e.g., “Is the person reaching”). These data were not normally distributed. Five participants (two OT, one AVP, and two placebo) had accuracy more than 2.5 standard deviations below the mean (i.e., accuracy below 76%). Excluding these participants did not change these results, and we report results without exclusions. No significant differences were found for mentalizing (i.e., Why task) for OT (*M*=90.6%, *SD*=8.6%) compared to placebo (*M*=91.5%, *SD*=6.5%)(*t_153_*=0.77, *p*=.443, 95% CI [-1.47%, 3.34%], *Cohen’s d*=0.124), or for AVP (*M*=91.0%, *SD*=5.5%) compared to placebo (*t_113_*=0.41, *p*=.683, 95% CI [-2.07%, 3.15%], *Cohen’s d*=0.086). Similarly, when engaged in action understanding (i.e., How task), no significant differences were found for OT (*M=*92.8%, *SD=*6.5%) compared to placebo (*M=*92.8%, *SD=*6.1%) (*t_153_*=0.003, *p*=.998, 95% CI [-2.01%, 2.02%], *Cohen’s d*=0.000), or for AVP (*M*=93.3%, *SD*=4.5%) compared to placebo *(t_113_*=0.409, *p*=.683, 95% CI [-1.90%, 2.89%], *Cohen’s d*=0.086).

***Reaction time***

Based on results showing differences in reaction time between Why and How tasks (Spunt & Adolphs, 2014), we examined whether either neuropeptide had an effect on reaction time when mentalizing or when engaged in action understanding. Analyses included both “correct” and “incorrect” trials. No significant differences were found in reaction time when mentalizing (i.e., Why task) for OT (*M*=0.852 s, *SD*=0.138 s) compared to placebo (*M*=0.835 s, *SD*=0.125 s) (*t_153_*=0.818, *p*=.415, 95% CI [-0.024 s, 0.059 s], *Cohen’s d*=0.132), or AVP (*M*=0.874 s, *SD*=0.224 s) compared to placebo (*t_113_=*1.188*, p=*.237*,* 95% CI [-0.026 s ,0.105 s], *Cohen’s d=*0.250). In addition, no significant differences were found in reaction time when engaged in action understanding (i.e., How task) for OT (*M*=0.776 s, *SD*=0.124 s) compared to placebo (*M*=0.758 s, *SD*=0.103 s) (*t_153_*=1.005, *p*=.316, 95% CI [-0.018 s ,0.054 s], *Cohen’s d*=0.162), or AVP (*M=*0.793 s*, SD=*0.186 s) compared to placebo (*t_113_*=1.271, *p*=.206, 95% CI [-0.019 s, 0.089 s], *Cohen’s d*=0.267). Given that Spunt and Adolphs (2014) found evidence that faster reaction time was related to better accuracy, we re-ran analyses including only “correct” trials. The results remained unchanged.

***Drug effects on changes in affect and state anxiety***

To examine whether any observed effects of drug condition could result from changes in affect or state anxiety, we analyzed non-specific main effects of drug condition (AVP, OT, placebo). A one-way ANOVA showed no pre-post changes in positive affect, *F* (2, 184)=.47, *p*=.63, η_p_^2^=.005, negative affect, *F* (2, 184)=1.08, *p*=.34, η_p_^2^=.012, or state anxiety *F* (2, 184)=.34, *p*=.71, η_p_^2^=.004. In addition, no significant differences were found between OT vs. placebo or AVP vs. placebo.

***Exploratory neural activation analyses***

Though the amygdala has not been associated with the Why versus How contrast, it is the region most consistently observed in either activation or deactivation following OT administration (Averbeck, 2010; Bartz, Zaki, Bolger, & Ochsner, 2011; Grace, Rossell, Heinrichs, Kordsachia, & Labuschagne, 2018; Wang, Yan, Li, & Ma, 2017; Zink & Meyer-Lindenberg, 2012), and it hosts a high concentration of OT receptors (Boccia, Petrusz, Suzuki, Marson, & Pedersen, 2013). Therefore, in an exploratory analysis, we included the amygdala as an ROI as well but found no significant effect for either OT (*t*_153_=0.35, *p*=.727, *Cohen’s d*=0.056) or AVP (*t*_113_=0.368, *p*=.714, *Cohen’s d*=0.077) compared to placebo.

**References**

Averbeck, B. B. (2010). Oxytocin and the salience of social cues. *Proceedings of the National Academy of Sciences of the United States of America*, *107*(20), 9033–9034. https://doi.org/10.1073/pnas.1004892107

Bagby, R. M., Parker, J. D. A., & Taylor, G. J. (1994). The twenty-item Toronto Alexithymia scale-I. Item selection and cross-validation of the factor structure. *Journal of Psychosomatic Research*, *38*(1), 23–32. https://doi.org/10.1016/0022-3999(94)90005-1

Baron-Cohen, S., & Wheelwright, S. (2004). The empathy quotient: An investigation of adults with asperger syndrome or high functioning autism, and normal sex differences. *Journal of Autism and Developmental Disorders*, *34*(2), 163–175. https://doi.org/10.1023/B:JADD.0000022607.19833.00

Baron-Cohen, S., Wheelwright, S., Skinner, R., Martin, J., & Clubley, E. (2001). The autism-spectrum quotient (AQ): evidence from Asperger syndrome/high-functioning autism, males and females, scientists and mathematicians. *Journal of Autism and Developmental Disorders*, *31*(1), 5–17. https://doi.org/10.1023/A:1005653411471

Bartz, J. A., Nitschke, J. P., Krol, S. A., & Tellier, P. P. (2019). Oxytocin Selectively Improves Empathic Accuracy: A Replication in Men and Novel Insights in Women. *Biological Psychiatry: Cognitive Neuroscience and Neuroimaging*, *4*(12), 1042–1048. https://doi.org/10.1016/j.bpsc.2019.01.014

Bartz, J. A., Zaki, J., Bolger, N., Hollander, E., Ludwig, N. N., Kolevzon, A., & Ochsner, K. N. (2010). Oxytocin selectively improves empathic accuracy. *Psychological Science*, *21*(10), 1426–1428. https://doi.org/0956797610383439 [pii] 10.1177/0956797610383439

Bartz, J. A., Zaki, J., Bolger, N., & Ochsner, K. N. (2011). Social effects of oxytocin in humans: context and person matter. *Trends in Cognitive Sciences*, *15*(7), 301–309. https://doi.org/10.1016/j.tics.2011.05.002

Boccia, M. L., Petrusz, P., Suzuki, K., Marson, L., & Pedersen, C. A. (2013). Immunohistochemical localization of oxytocin receptors in human brain. *Neuroscience*, *253*, 155–164. https://doi.org/10.1016/j.neuroscience.2013.08.048

Brunnlieb, C., Münte, T. F., Tempelmann, C., & Heldmann, M. (2013). Vasopressin modulates neural responses related to emotional stimuli in the right amygdala. *Brain Research*, *1499*, 29–42. https://doi.org/10.1016/j.brainres.2013.01.009

Chen, X., Gautam, P., Haroon, E., & Rilling, J. K. (2017). Within vs. between-subject effects of intranasal oxytocin on the neural response to cooperative and non-cooperative social interactions. *Psychoneuroendocrinology*, *78*, 22–30. https://doi.org/10.1016/j.psyneuen.2017.01.006

Costa, P. T., & McCrae, R. R. (1992). Revised NEO Personality Inventory (NEO-PIR) and NEO Five Factor Inventory (NEO-FFI) professional manual. *Psychological Assessment Resources*. Odessa, FL.

Davis, M. H. (1983). Measuring individual differences in empathy: Evidence for a multidimensional approach. *Journal of Personality and Social Psychology*, *44*(1), 113–126. https://doi.org/10.1037/0022-3514.44.1.113

Feeser, M., Fan, Y., Weigand, A., Hahn, A., Gärtner, M., Böker, H., … Bajbouj, M. (2015). Oxytocin improves mentalizing - pronounced effects for individuals with attenuated ability to empathize. *Psychoneuroendocrinology*, *53*, 223–232. https://doi.org/10.1016/j.psyneuen.2014.12.015

Feng, C., Hackett, P. D., DeMarco, A. C., Chen, X., Stair, S., Haroon, E., … Rilling, J. K. (2015). Oxytocin and vasopressin effects on the neural response to social cooperation are modulated by sex in humans. *Brain Imaging and Behavior*, *9*(4), 754–764. https://doi.org/10.1007/s11682-014-9333-9

Geng, Y., Zhao, W., Zhou, F., Ma, X., Yao, S., Hurlemann, R., … Kendrick, K. M. (2018). Oxytocin Enhancement of Emotional Empathy: Generalization Across Cultures and Effects on Amygdala Activity. *Frontiers in Neuroscience*, *12*, 512. https://doi.org/10.3389/fnins.2018.00512

Grace, S. A., Rossell, S. L., Heinrichs, M., Kordsachia, C., & Labuschagne, I. (2018, October). Oxytocin and brain activity in humans: A systematic review and coordinate-based meta-analysis of functional MRI studies. *Psychoneuroendocrinology*, *96*, 6–24. https://doi.org/10.1016/j.psyneuen.2018.05.031

Groppe, S. E., Gossen, A., Rademacher, L., Hahn, A., Westphal, L., Gründer, G., & Spreckelmeyer, K. N. (2013). Oxytocin influences processing of socially relevant cues in the ventral tegmental area of the human brain. *Biological Psychiatry*, *74*(3), 172–179. https://doi.org/10.1016/j.biopsych.2012.12.023

Human, L. J., Thorson, K. R., & Mendes, W. B. (2016). Interactive Effects Between Extraversion and Oxytocin Administration. *Social Psychological and Personality Science*, *7*(7), 735–744. https://doi.org/10.1177/1948550616644964

Iacoboni, M. (2009). Imitation, Empathy, and Mirror Neurons. *Annual Review of Psychology*, *60*(1), 653–670. https://doi.org/10.1146/annurev.psych.60.110707.163604

Liebowitz, M. R. (1987). Social phobia. *Modern Problems in Pharmacopsychiatry*, *22*, 141–173. https://doi.org/10.1159/000414022

Luminet, O., Grynberg, D., Ruzette, N., & Mikolajczak, M. (2011). Personality-dependent effects of oxytocin: Greater social benefits for high alexithymia scorers. *Biological Psychology*, *87*(3), 401–406. https://doi.org/10.1016/j.biopsycho.2011.05.005

Mattick, R. P., Clark, J. C., & Clarke, J. C. (1998). Development and validation of measures of social phobia scrutiny fear and social interaction anxiety. *Behaviour Research and Therapy*, *36*(4), 455–470. https://doi.org/10.1016/S0005-7967(97)10031-6

Niedenthal, P. M., Mermillod, M., Maringer, M., & Hess, U. (2010). The Simulation of Smiles (SIMS) model: Embodied simulation and the meaning of facial expression. *Behavioral and Brain Sciences, 33*(6)*,* 417-433. https://doi.org/10.1017/S0140525X10000865

Parker, G., Tupling, H., & Brown, L. B. (1979). A Parental Bonding Instrument. *British Journal of Medical Psychology*, *52*(1), 1–10. https://doi.org/10.1111/j.2044-8341.1979.tb02487.x

Preston, S. D., & de Waal, F. B. M. (2002). Empathy: Its ultimate and proximate bases. *Behavioral and Brain Sciences*, *25*(1), 1–20. https://doi.org/10.1017/S0140525X02000018

Radke, S., & de Bruijn, E. R. A. (2015). Does oxytocin affect mind-reading? A replication study. *Psychoneuroendocrinology*, *60*, 75–81. https://doi.org/10.1016/j.psyneuen.2015.06.006

Riem, M. M. E., Bakermans-Kranenburg, M. J., Voorthuis, A., & van IJzendoorn, M. H. (2014). Oxytocin effects on mind-reading are moderated by experiences of maternal love withdrawal: An fMRI study. *Progress in Neuro-Psychopharmacology and Biological Psychiatry*, *51*, 105–112. https://doi.org/10.1016/j.pnpbp.2014.01.014

Schwaiger, M., Heinrichs, M., & Kumsta, R. (2019). Oxytocin administration and emotion recognition abilities in adults with a history of childhood adversity. *Psychoneuroendocrinology*, *99*, 66–71. https://doi.org/10.1016/j.psyneuen.2018.08.025

Spielberger, C. D., Gorsuch, R. L., Lushene, R., Vagg, P. R., & Jacobs, G. A. (1983). *Manual for the State-Trait Anxiety Inventory.* Palo Alto, CA: Consulting Psychologists Press.

Spunt, R. P., & Adolphs, R. (2014). Validating the Why/How contrast for functional MRI studies of Theory of Mind. *NeuroImage*, *99*, 301–311. https://doi.org/10.1016/j.neuroimage.2014.05.023

Tabak, B. A., Meyer, M. L., Castle, E., Dutcher, J. M., Irwin, M. R., Han, J. H., … Eisenberger, N. I. (2015). Vasopressin, but not oxytocin, increases empathic concern among individuals who received higher levels of paternal warmth: A randomized controlled trial. *Psychoneuroendocrinology*, *51*, 253–261. https://doi.org/10.1016/j.psyneuen.2014.10.006

Tabak, B. A., Meyer, M. L., Dutcher, J. M., Castle, E., Irwin, M. R., Lieberman, M. D., & Eisenberger, N. I. (2016). Oxytocin, but not vasopressin, impairs social cognitive ability among individuals with higher levels of social anxiety: A randomized controlled trial. *Social Cognitive and Affective Neuroscience*, *11*(8), 1272–1279. https://doi.org/10.1093/scan/nsw041

Taylor, S. E., Lerner, J. S., Sage, R. M., Lehman, B. J., & Seeman, T. E. (2004). Early environment, emotions, responses to stress, and health. *Journal of Personality*, *72*(4), 1365–1393. https://doi.org/10.1111/j.1467-6494.2004.00300.x

Teed, A. R., Han, K., Rakic, J., Mark, D. B., & Krawczyk, D. C. (2019). The influence of oxytocin and vasopressin on men’s judgments of social dominance and trustworthiness: An fMRI study of neutral faces. *Psychoneuroendocrinology*, *106*, 252–258. https://doi.org/10.1016/j.psyneuen.2019.04.014

Thompson, E. R. (2007). Development and Validation of an Internationally Reliable Short-Form of the Positive and Negative Affect Schedule (PANAS). *Journal of Cross-Cultural Psychology*, *38*(2), 227–242. https://doi.org/10.1177/0022022106297301

Tomova, L., Heinrichs, M., & Lamm, C. (2019). The Other and Me: Effects of oxytocin on self-other distinction. *International Journal of Psychophysiology*, *136*, 49–53. https://doi.org/10.1016/j.ijpsycho.2018.03.008

Wakabayashi, A., Baron-Cohen, S., Wheelwright, S., Goldenfeld, N., Delaney, J., Fine, D., … Weil, L. (2006). Development of short forms of the Empathy Quotient (EQ-Short) and the Systemizing Quotient (SQ-Short). *Personality and Individual Differences*, *41*(5), 929–940. https://doi.org/10.1016/j.paid.2006.03.017

Wang, D., Yan, X., Li, M., & Ma, Y. (2017). Neural substrates underlying the effects of oxytocin: a quantitative meta-analysis of pharmaco-imaging studies. *Social Cognitive and Affective Neuroscience*, *12*(10), 1565–1573. https://doi.org/10.1093/scan/nsx085

Zhao, W., Yao, S., Li, Q., Geng, Y., Ma, X., Luo, L., … Kendrick, K. M. (2016). Oxytocin blurs the self-other distinction during trait judgments and reduces medial prefrontal cortex responses. *Human Brain Mapping*, *37*(7), 2512–2527. https://doi.org/10.1002/hbm.23190

Zink, C. F., & Meyer-Lindenberg, A. (2012). Human neuroimaging of oxytocin and vasopressin in social cognition. *Hormones and Behavior*, *61*(3), 400–409. https://doi.org/10.1016/j.yhbeh.2012.01.016

**Supplementary Table S1**

*Self-report measures included in exploratory moderation analyses of OT and AVP effects*

| **Measure** | **Construct** | **α** | **Reference** |
| --- | --- | --- | --- |
| Autism Spectrum Quotient | Traits related to autism | .69 | (Baron-Cohen et al., 2001) |
| Empathy Quotient -Short | Trait empathy | .91 | (Wakabayashi et al., 2006) |
| Interpersonal Reactivity Index | Trait empathy |  | (Davis, 1983) |
| Empathic Concern | Affective empathy | .79 |  |
| Perspective Taking | Cognitive empathy | .81 |  |
| NEO Five Factor Inventory | Personality traits |  | (Costa & McCrae, 1992) |
| Extraversion | Extraversion | .77 |  |
| Agreeableness | Agreeableness | .76 |  |
| Parental Bonding Instrument | Parental warmth |  | (Parker et al., 1979) |
| Maternal Warmth | Maternal warmth in childhood | .92 |  |
| Paternal Warmth | Paternal warmth in childhood | .93 |  |
| Risky Families Questionnaire | Family adversity in childhood | .86 | (Taylor et al., 2004) |
| Toronto Alexithymia Scale | Alexithymia | .84 | (Bagby, Parker, & Taylor, 1994) |
| Social Phobia Scale | Social anxiety | .92 | (Mattick, Clark, & Clarke, 1998) |
| Social Interaction Anxiety Scale | Social anxiety | .89 | (Mattick et al., 1998) |
| Liebowitz Social Anxiety Scale | Social anxiety | .95 | (Liebowitz, 1987) |

*Note*. Eleven participants did not have a father present as a young child, thus, *n*=175 for our measure of paternal warmth. As in our previous work (Tabak et al., 2016), we created a composite measure of social anxiety that included standardized and averaged scores from the three social anxiety scales described above (α=.88).

**Supplementary Table S2**

*Differences between OT or AVP vs. placebo on accuracy and reaction time based on gender*

| **Contrast** | **Null Hypothesis Significance Testing** | | | | | | | **Bayes Factor** | |  |
| --- | --- | --- | --- | --- | --- | --- | --- | --- | --- | --- |
|  | df | t-value | *p-* value | *p*-value FDR | Upper  95% CI | Lower 95% CI | *Cohen’s d* | BF01 | BF10 |  |
|  |  |  |  |  |  |  |  |  |  |  |
| Males | | | | | | | | | |  |
| Acc Why OT vs PL | 65 | 0.401 | 0.689 | 0.919 | -3.16 | 4.74 | 0.098 | 5.02 | 0.199 |  |
| Acc How  OT vs PL | 65 | 0.84 | 0.404 | 0.715 | -1.95 | 4.79 | 0.205 | 3.91 | 0.256 |  |
| RT Why OT vs PL | 65 | 0.817 | 0.417 | 0.803 | -0.044 | 0.106 | 0.2 | 5.07 | 0.197 |  |
| RT How OT vs PL | 65 | 1.123 | 0.266 | 0.715 | -0.027 | 0.095 | 0.274 | 4.26 | 0.235 |  |
| Females | | | | | | | | | |  |
| Acc Why  OT vs PL | 86 | 0.623 | 0.535 | 0.715 | -2.11 | 4.03 | 0.134 | 4.16 | 0.24 |  |
| Acc How OT vs PL | 86 | 0.874 | 0.385 | 0.963 | -1.41 | 3.61 | 0.188 | 5.74 | 0.174 |  |
| Acc Why AVP vs PL | 79 | 0.835 | 0.406 | 0.715 | -0.043 | 0.052 | 0.191 | 3.98 | 0.251 |  |
| Acc How AVP vs PL | 79 | 0.047 | 0.963 | 0.715 | -0.04 | 0.049 | 0.011 | 3.04 | 0.329 |  |
| RT Why OT vs PL | 86 | 0.185 | 0.854 | 0.932 | -1.24 | 3.04 | 0.04 | 5.98 | 0.167 |  |
| RT How OT vs PL | 86 | 0.198 | 0.844 | 0.932 | -1.94 | 2.03 | 0.043 | 5.96 | 0.168 |  |
| RT Why AVP vs PL | 79 | 1.151 | 0.253 | 0.715 | -0.032 | 0.119 | 0.263 | 3.12 | 0.32 |  |
| RT How AVP vs PL | 79 | 1.075 | 0.286 | 0.715 | -0.03 | 0.099 | 0.246 | 3.37 | 0.297 |  |

*Note.* RT=Reaction time, Acc = Accuracy, OT = Oxytocin, AVP = Vasopressin, PL = Placebo.

**Supplementary Figure S1**

*CONSORT Flow Chart*

**Supplementary Figure S2**

*Study timeline*


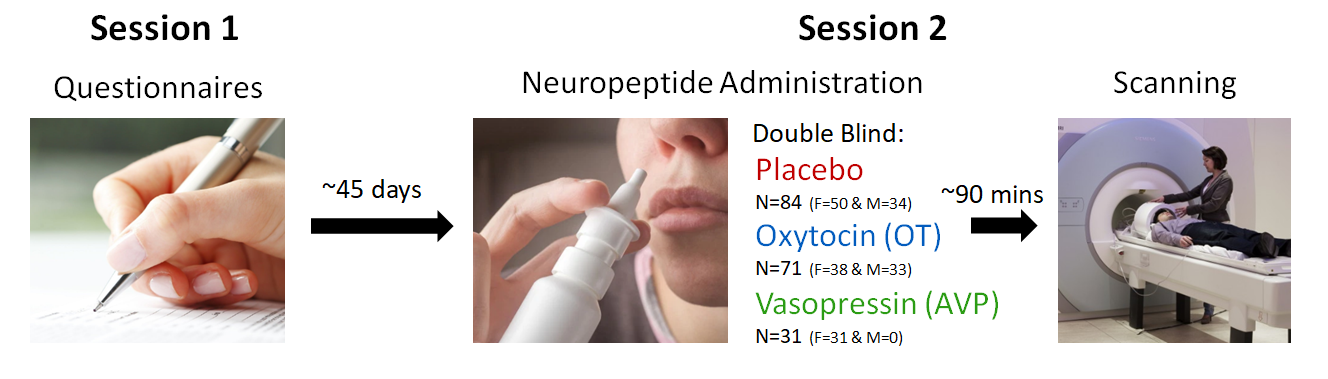


*Note.* In session 1, participants filled out questionnaires about themselves. Approximately 45 days later, they came into the lab and were randomly assigned to receive either placebo, OT, or AVP. On average, 40 minutes later they were put into the fMRI scanner to begin a series of tasks with the Why/How task approximately 90 minutes after the neuropeptide administration.

**Supplementary Figure S3**

*Equivalence Tests for neural and behavioral effects*


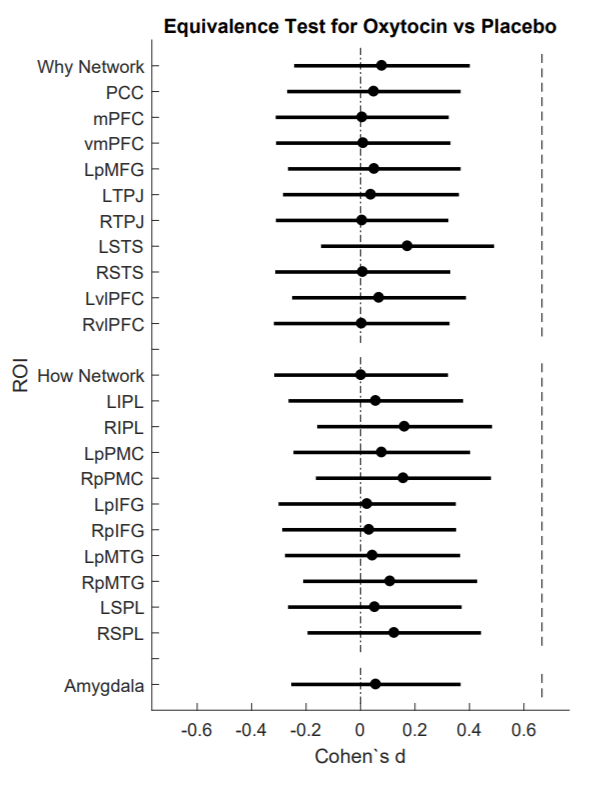

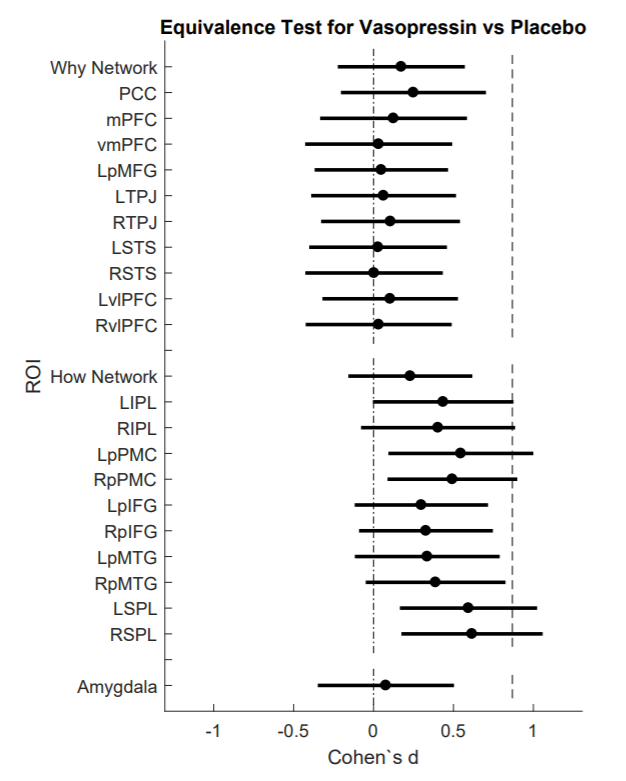


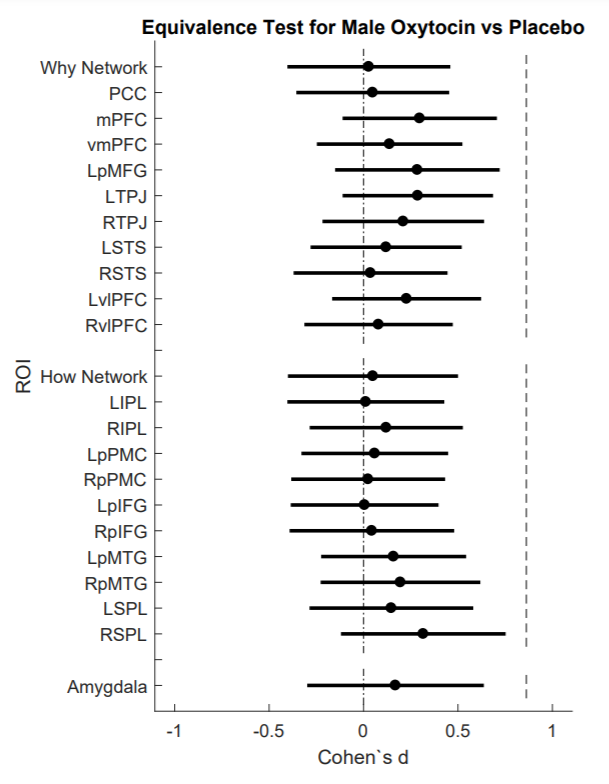

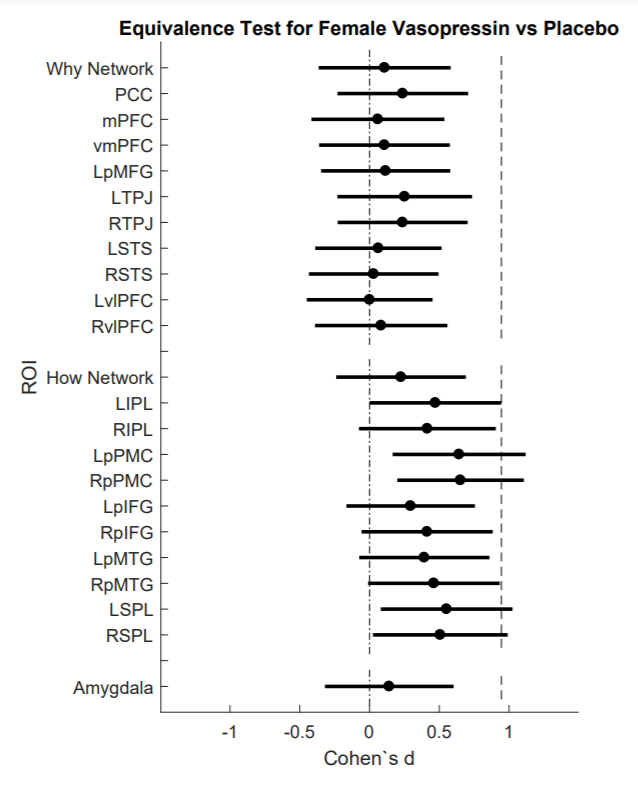


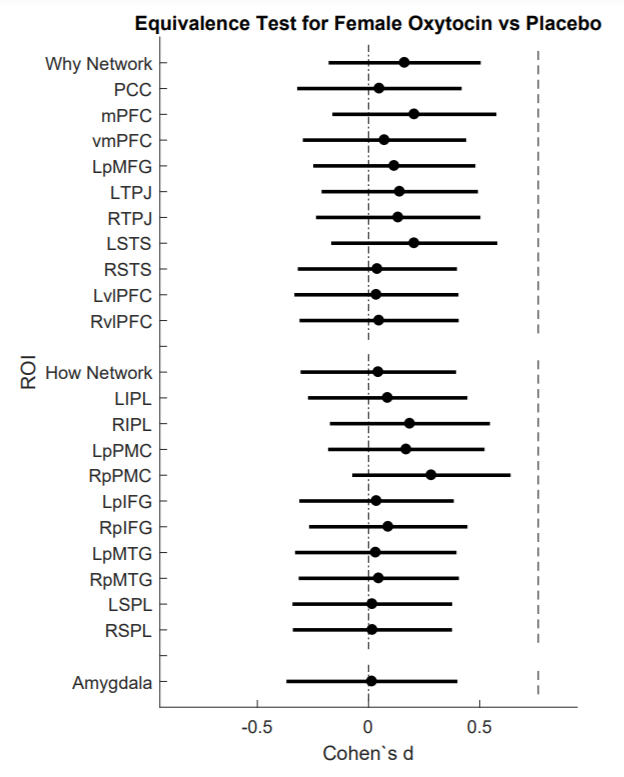

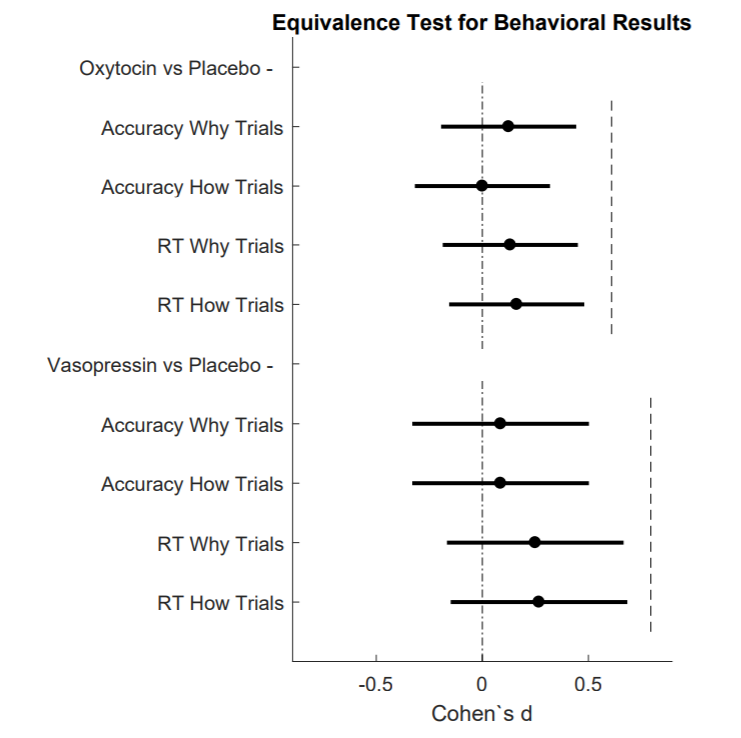


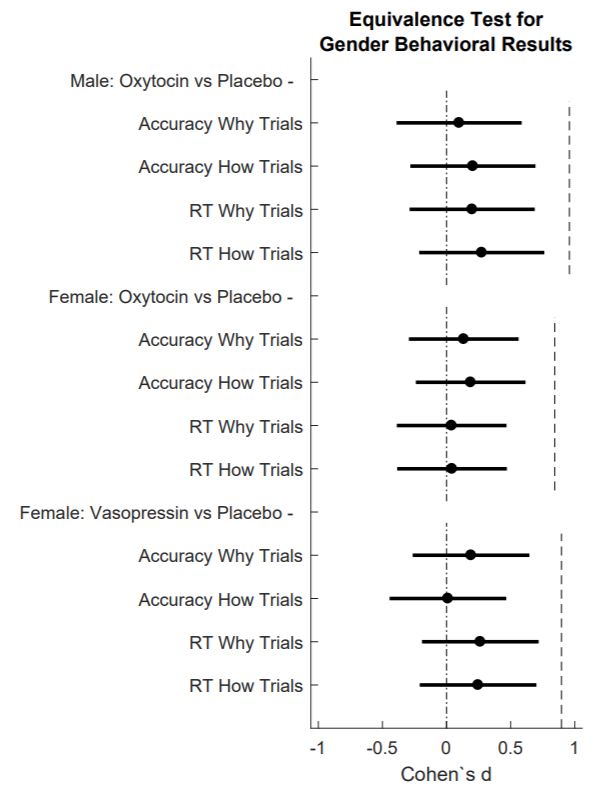


*Note.* The dark bars are the 95% confidence Cohen’s d values for each contrast. The dashed line represents upper bound for equivalence testing taking into account the sample size of the contrast and Bonferroni corrected threshold p-value to account for the multiple comparisons.

**Supplementary Figure S4**

*Differences between OT, AVP, and placebo in accuracy and reaction time on the Why/How task based on gender*

a)


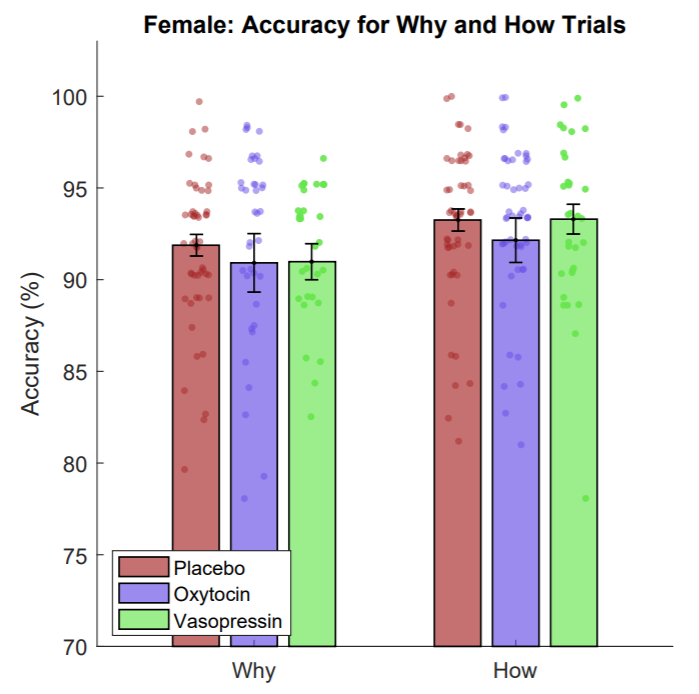

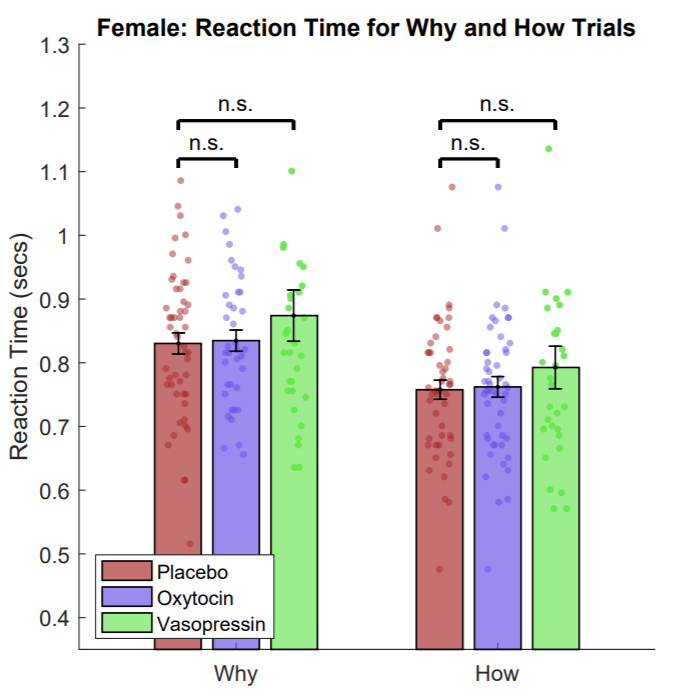


b)


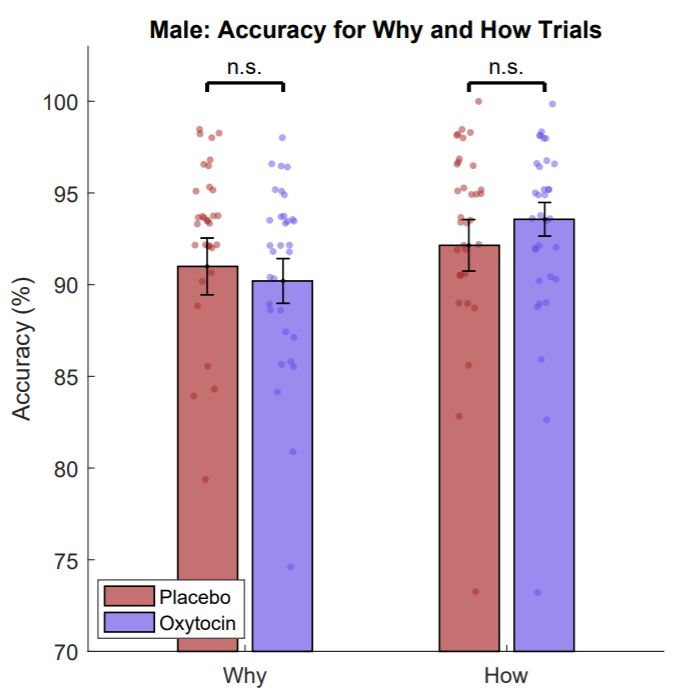

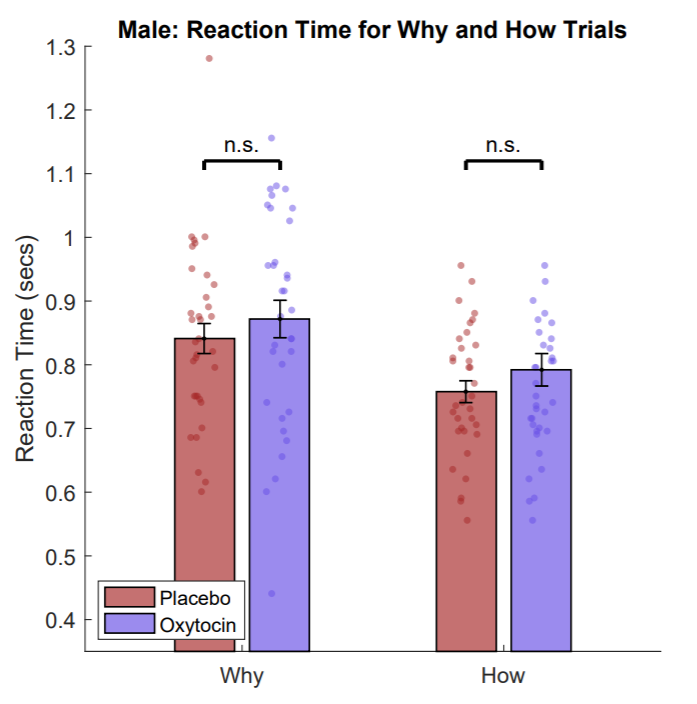


*Note.* No significant behavioral differences for the Why and How task in relation to OT or AVP vs. placebo when broken down by gender. Since AVP was only given to the females, there is no male vasopressin contrast. Error bars represent standard error. n.s.= not significant.

**Supplementary Figure S5**

*Neural Activation of Drug Condition for Why vs How based on gender*

***Female Oxytocin Male Oxytocin***


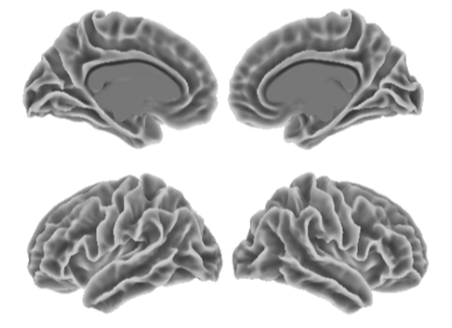

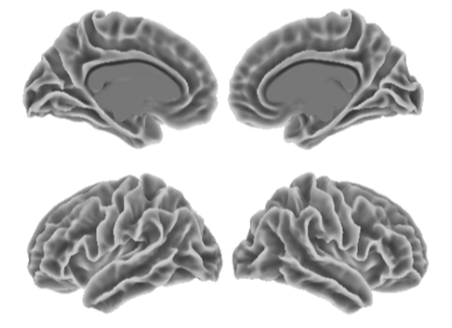


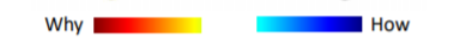

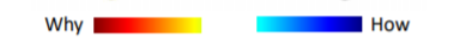


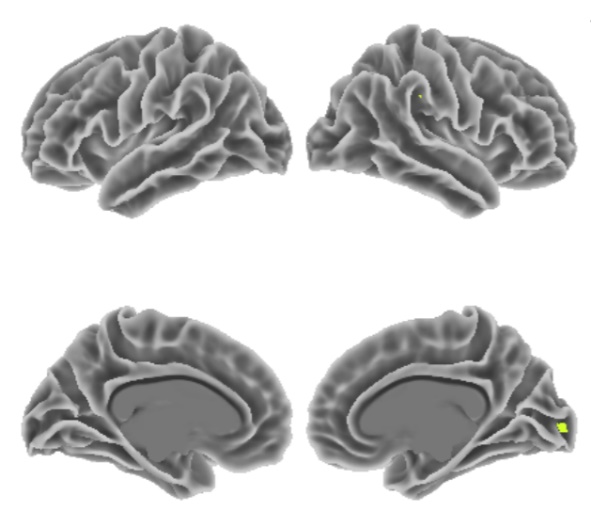

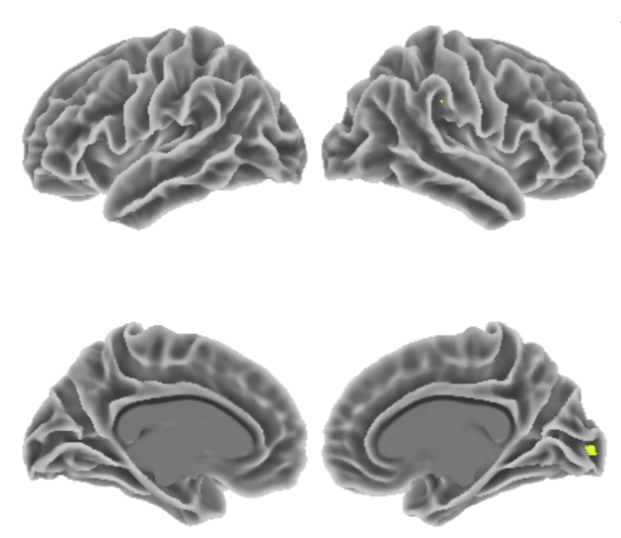
 ***Female Vasopressin***


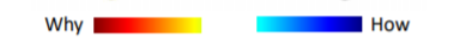


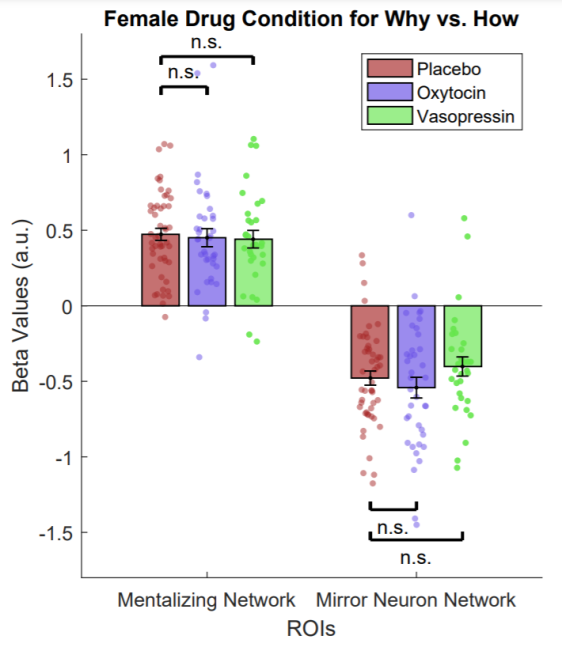

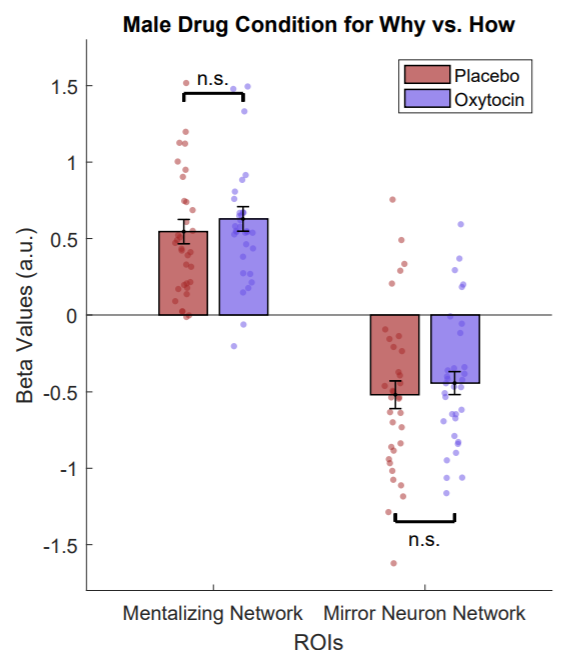


**Supplementary Figure S6**

*Changes in functional connectivity when separating the sample based on gender*


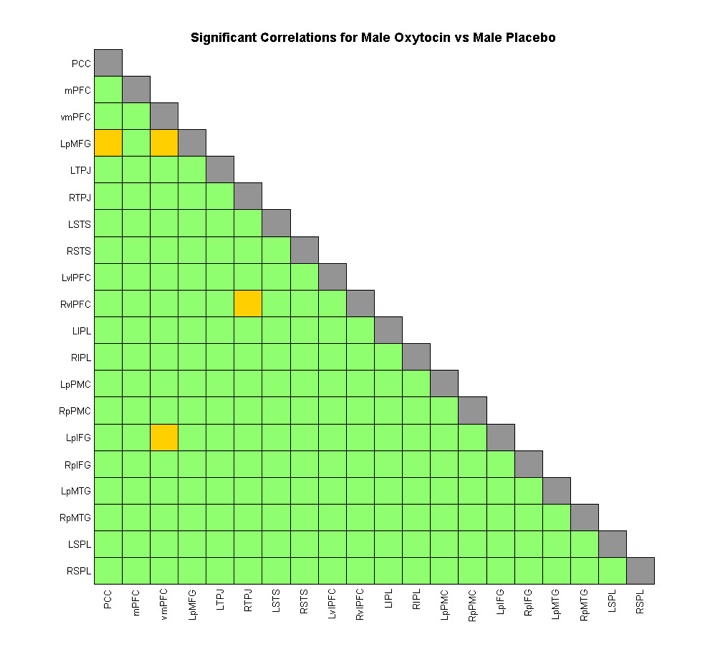

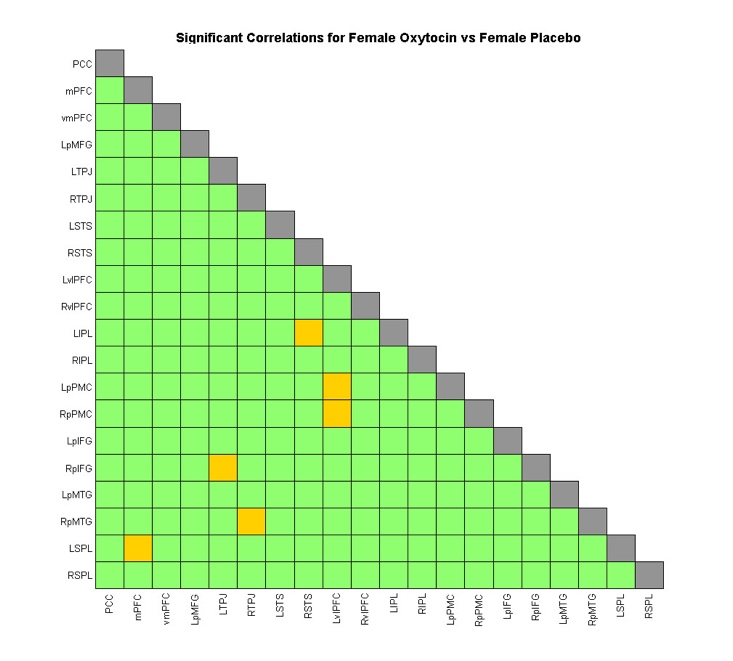


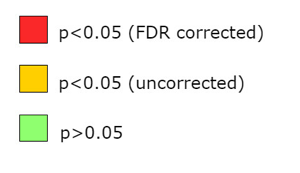


*Note.* PCC = Posterior Cingulate Cortex/Precuneus, mPFC = medial and dorsal medial Prefrontal Cortex, vmPFC = ventromedial Prefrontal Cortex, LpMFG = Left posterior Middle Frontal Gyrus, LTPJ = Left Temporoparietal Junction, RTPJ = Right Temporoparietal Junction, RSTS = Right Superior Temporal Sulcus, LSTS = Left Superior Temporal Sulcus, LvlPFC = Left ventral lateral Prefrontal Cortex, RvlPFC = Right ventral lateral Prefrontal Cortex, LIPL = Left Intraparietal Lobule, RIPL = Right Intraparietal Lobule, LpPMC = Left posterior Premotor Cortex, RpPMC = Right posterior Premotor Cortex, LpIFG = Left posterior Inferior Frontal Gyrus, RpIFG = Right posterior Inferior Frontal Gyrus, LpMTG = Left posterior Middle Temporal Gyrus, RpMTG = Right posterior Middle Temporal Gyrus, LSPL = Left Superior Parietal Lobule, and RSPL = Right Superior Parietal Lobule.

**Supplementary Figure S7**

*Interaction of OT or AVP vs. placebo with relevant individual differences*


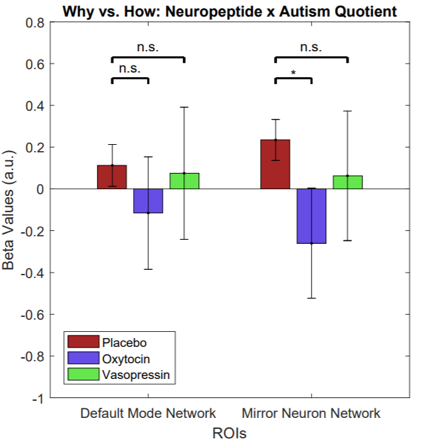

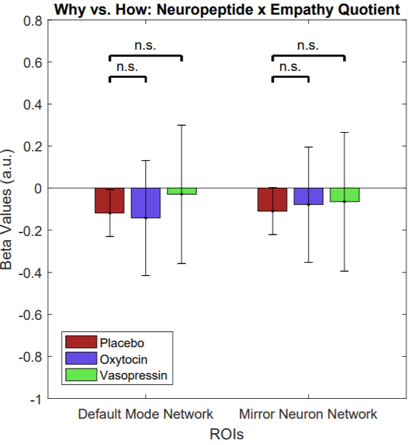

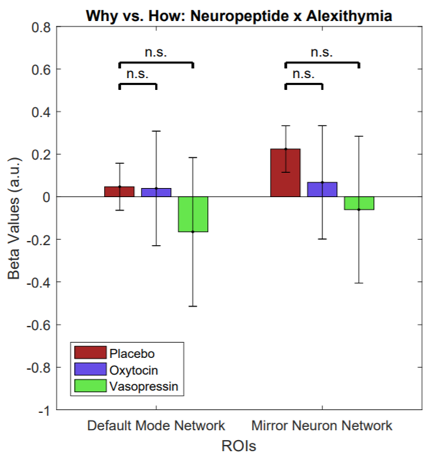


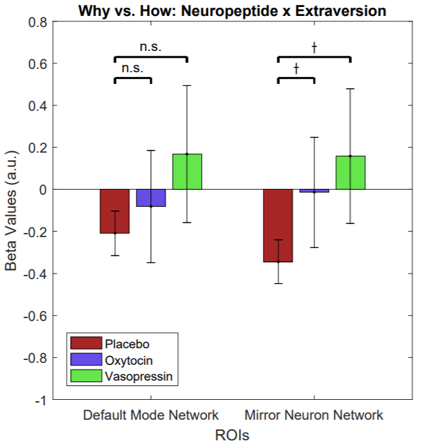

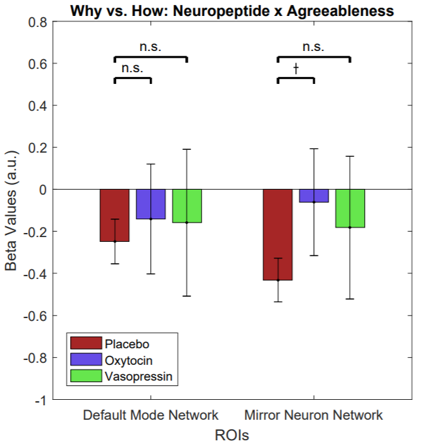

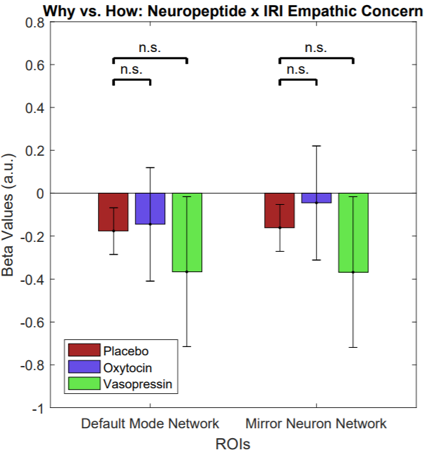


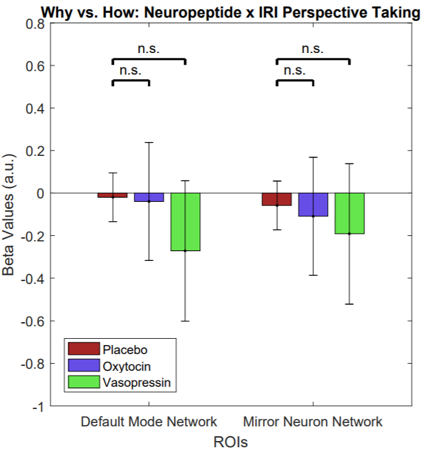

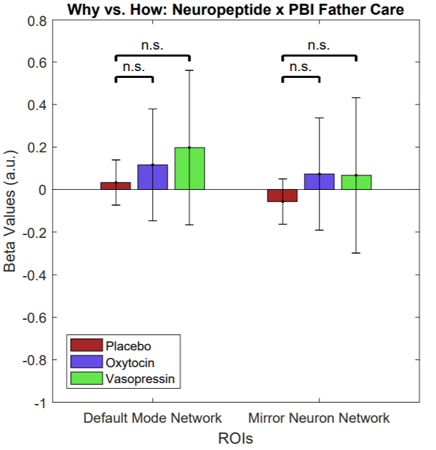

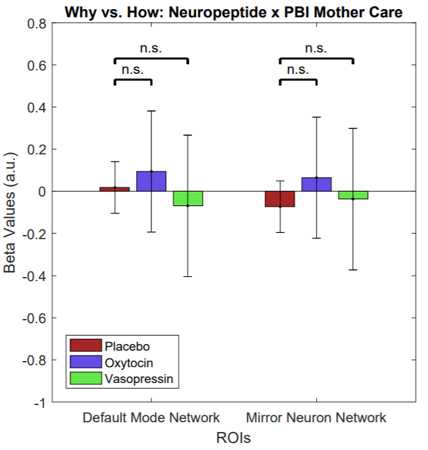


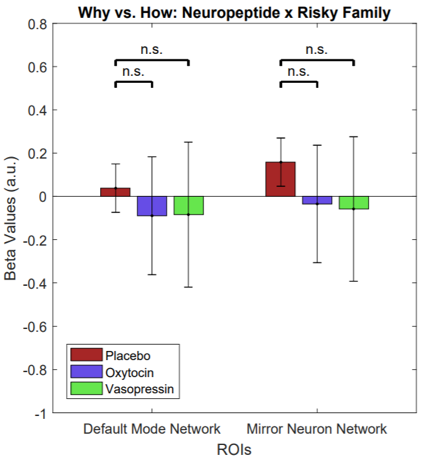

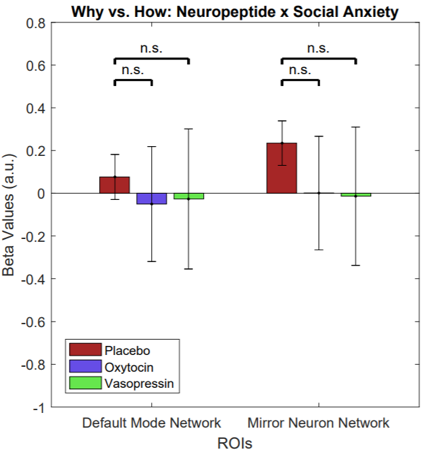


*Note.* Higher values represent more activation for Why trials and lower values represent more activation for How trials. In order to account for the 44 comparisons, while still being sensitive to effects, only p-values smaller than 0.01 (uncorrected) are considered significant. Error bars represent standard error. *p*<.001**, *p*<.01*, *p*<.05♱, n.s.= not significant.
